# Synthesis of Giant Dendritic Polyphenylenes with 366 and 546 Carbon Atoms and Their High‐vacuum Electrospray Deposition

**DOI:** 10.1002/asia.202200220

**Published:** 2022-04-22

**Authors:** Ian Cheng‐Yi Hou, Antoine Hinaut, Sebastian Scherb, Ernst Meyer, Akimitsu Narita, Klaus Müllen

**Affiliations:** ^1^ Synthetic Chemistry Max Planck Institute for Polymer Research Ackermannweg 10 D-55128 Mainz Germany; ^2^ Department of Chemistry Johannes Gutenberg University Mainz Duesbergweg 10–14 D-55128 Mainz Germany; ^3^ Department of Physics University of Basel Klingelbergstrasse 82 4056 Basel Switzerland

**Keywords:** polyphenylene, dendrimer, Diels-Alder reaction, electrospray, nanographene

## Abstract

Dendritic polyphenylenes (PPs) can serve as precursors of nanographenes (NGs) if their structures represent 2D projections without overlapping benzene rings. Here, we report the synthesis of two giant dendritic PPs fulfilling this criteria with 366 and 546 carbon atoms by applying a “layer‐by‐layer” extension strategy. Although our initial attempts on their cyclodehydrogenation toward the corresponding NGs in solution were unsuccessful, we achieved their deposition on metal substrates under ultrahigh vacuum through the electrospray technique. Scanning probe microscopy imaging provides valuable information on the possible thermally induced partial planarization of such giant dendritic PPs on a metal surface.

## Graphical abstract

**Extremely large dendritic polyphenylenes** are synthesized and imaged by scanning probe microscopy after deposition on metal substrates under ultrahigh vacuum through the electrospray technique.

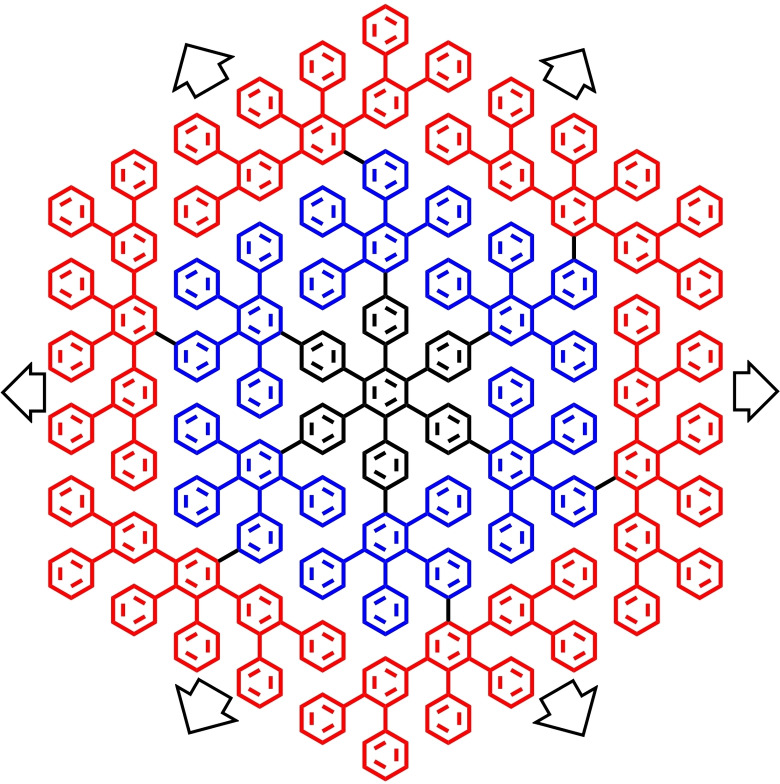

Dendritic polyphenylenes (PP) constitute a unique class of three‐dimensional macromolecules with scaffolds consisting of twisted, tightly interlocked benzene units.[^1^, ^2^, ^3^] Their semi‐rigid quasi‐spherical nanostructures, high intramolecular free volume, and outstanding thermal stability allow applications in various fields, including imaging, sensing, and diagnostics.[^4^, ^5^, ^6^] Moreover, dendritic PPs can serve as precursors for nanographenes (NGs) of defined size, shape, and edge structure.[^7^, ^8^, ^9^] Especially, NGs with sizes of 5–10 nm hold promise for exhibiting high charge carrier mobilities while still maintaining an energy gap that is essential for high‐performance transistors.[^10^, ^11^] However, synthesis of such large NGs as well as their precursors has remained challenging.

Previously, extremely large quasi‐spherical PP dendrimers have been achieved with a diameter above 30 nm.^[14]^ The synthesis of such PP dendrimers typically proceeds through (layer‐by‐layer) radial extension of a core by Diels‐Alder (D−A) reactions, using AB_2_‐type branching reagents. These building blocks are normally based on 2,3,4,5‐tetraphenylcyclopenta‐2,4‐diene‐1‐one (CP) derivatives, where the CP moiety serves as the diene (A) and two ethynyl groups as the dienophiles (B_2_). Nevertheless, the generally overcrowded structures of such PP dendrimers hinder their conversion into flat NGs.^[15]^ The typical requirement for PPs to serve as NG precursors is thus their structural representation in a 2D projection without spatially overlapping benzene rings (Figure 1). A representative example is the synthesis of *D*_6h_‐symmetric hexa‐*peri*‐hexabenzocoronene via oxidative cyclodehydrogenation of hexaphenylbenzene (HPB **1**) (Figure 1b).^[16]^ Employing dendritic PPs fulfilling this criterion, a homologous series of NGs with diameters of 1–3 nm has been achieved, exhibiting a correlation of size and molecular orbital energy gaps.[^17^, ^18^] The largest monodisperse NG synthesized to date is the so‐called C222 with 222 sp^2^ carbons in its hexagonal aromatic core and a diameter just exceeding 3 nm.[^12^, ^17^] The NG C222 is derived from the oxidative cyclodehydrogenation of dendritic PP **2** (Figure 1b).

**Figure 1:**
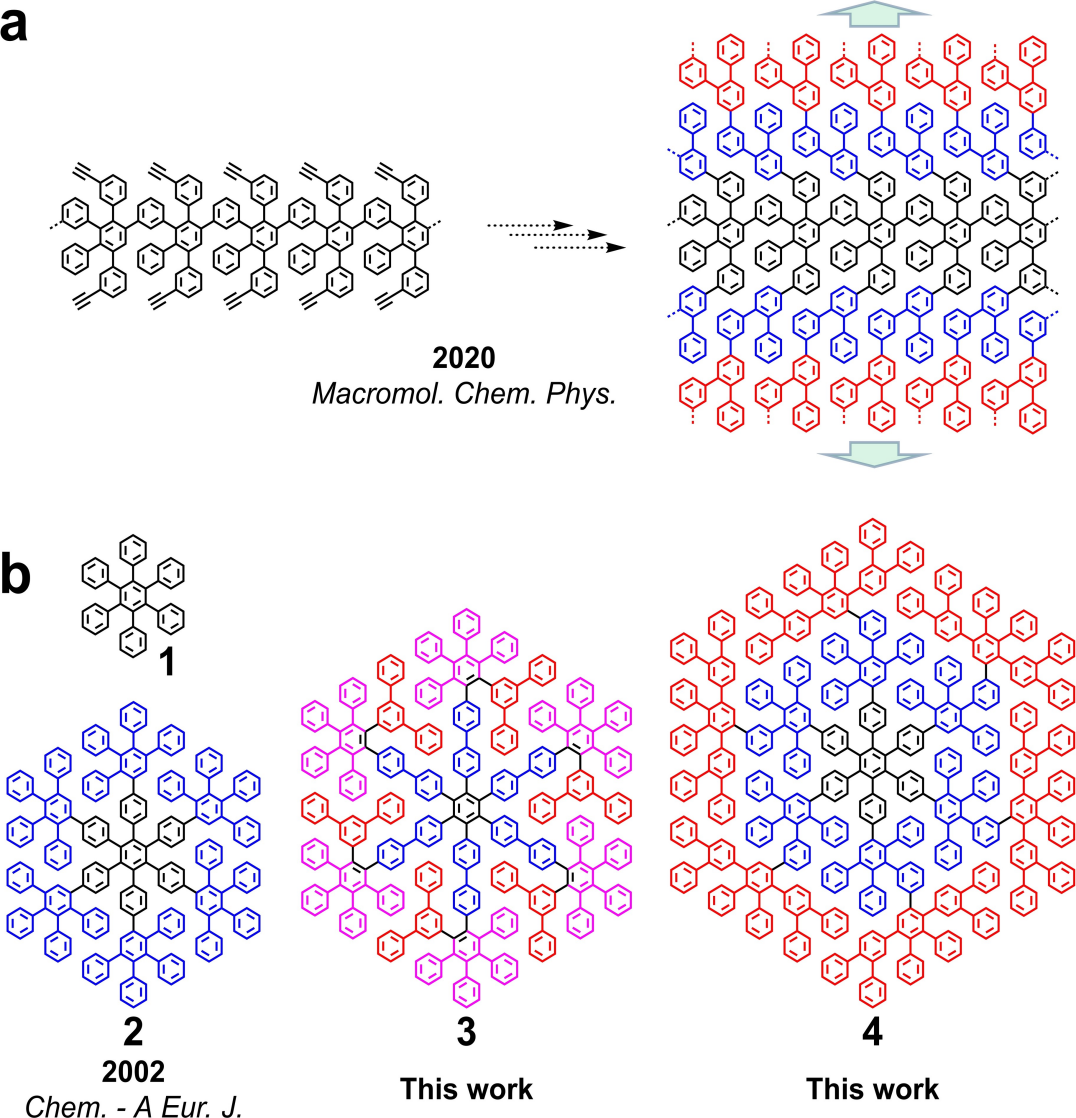
Layer‐by‐layer extension from (a) a linear PP and (b) an HPB core toward dendritic PPs as suitable precursors for NGs.[^12^, ^13^]

After the accomplishment of PP **2** and C222 in 2002, our group has been devoting attention to the synthesis of larger dendritic PPs as suitable precursors for NGs with a size approaching 5 nm.[^15^, ^19^, ^20^] Aside from these solution‐based syntheses, sublimation of dendritic PPs onto metal surfaces followed by thermal cyclodehydrogenation under ultra‐high vacuum (UHV) conditions can also furnish NGs.^[21]^ PPs with a size close to that of HPB **1** have already been successfully utilized for the on‐surface fabrication of NGs.[^22^, ^23^] Applying this protocol to larger dendritic PPs is, however, limited by their high sublimation temperatures.

Recently, we reported a series of laterally extended linear PPs as potential precursors for graphene nanoribbons with a width approaching 5 nm (Figure 1a).[^6^, ^13^] In their 2D projections, the linear PP backbones were “laterally” extended in a “layer‐by‐layer” fashion (Figure 1a). Here, we applied a similar layer‐by‐layer extension strategy to synthesize dendritic PP **4** by “radial” growth from a HPB core (Figure 1b). We have also synthesized dendritic PP **3** with a size between that of **2** and **4** by firstly constructing precursor **9** with a star‐shaped skeleton followed by its D−A reaction with six CPs (Figure 1b and Scheme 1). After the D−A reaction, the six outer *m*‐terphenylene units (indicated in red in Scheme 1) of **9** “backfold” onto the HPB core to fill the “voids” between biphenyl‐4,4′‐diyl moieties (indicated in blue in Scheme 1). Dendritic PPs **3** and **4** with 366 and 546 sp^2^ carbons, respectively, are potential precursors for giant NGs with a *D*_6h_ ‐symmetry and a diameter approaching 5 nm (Figure 1b). Compounds **3** and **4** were characterized by ^1^H NMR, ^13^C NMR, ^1^H NMR diffusion ordered spectroscopy (DOSY), and matrix‐assisted laser desorption/ionization time‐of‐flight (MALDI‐TOF) mass spectrometry (MS). Moreover, we carried out high‐vacuum electrospray deposition (HV‐ESD) of PPs **3** and **4** on metal substrates with the perspective of their on‐surface planarization, and achieved their visualization by *in‐situ* non‐contact atomic force microscopy (nc‐AFM) under UHV.

**Scheme 1:**
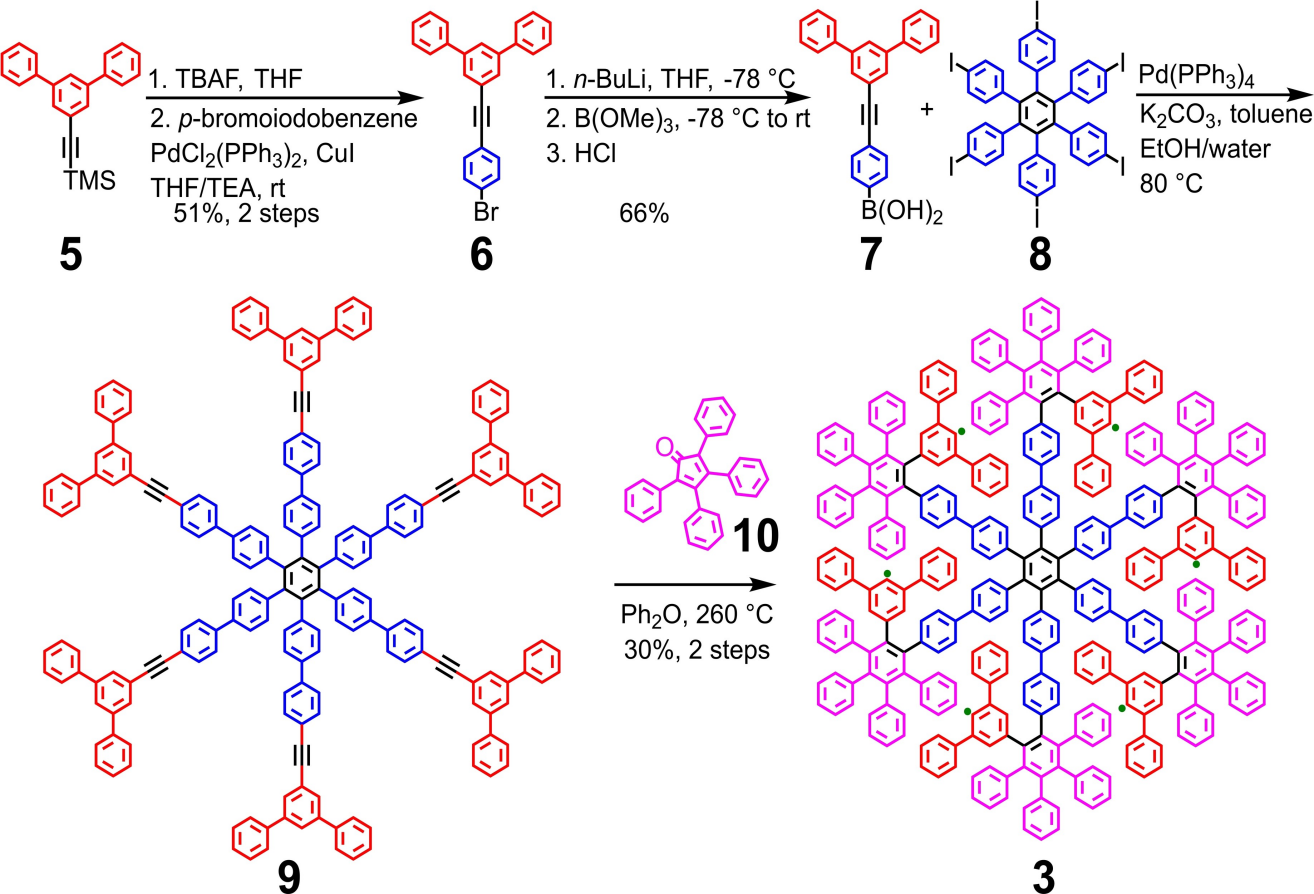
Synthesis of dendritic PP **3**. TBAF: tetrabutylammonium fluoride. THF: tetrahydrofuran. TEA: triethylamine. *n*‐BuLi: *n*‐butyllithium.

The synthesis of skeleton **9** started from 1‐(trimethylsilylethynyl)‐3,5‐diphenylbenzene (**5**)^[24]^ (Scheme 1). The trimethylsilyl protecting group of **5** was removed by treatment with tetrabutylammonium fluoride. The ethynyl group was then allowed to react with *p*‐bromoiodobenzene through a selective Sonogashira coupling at room temperature to afford 1‐(4‐bromophenylethynyl)‐3,5‐diphenylbenzene (**6**). Next, **6** was lithiated with *n*‐butyllithium and then converted to boronic acid **7** by nucleophilic attack on methyl borate followed by hydrolysis. Subsequently, a six‐fold Suzuki reaction between **7** and hexaiodo‐substituted HPB **8** ^[25]^ provided **9**, which was confirmed by MALDI‐TOF MS. The star‐shaped skeleton **9** exhibited poor solubility in common organic solvents, hindering thorough purification. Thus, the crude material of **9** was directly subjected to the six‐fold D−A reaction with pristine CP **10**. During the reaction at 260 °C, a suspension of **9** in the diphenyl ether solution of CP **10** gradually transformed into a transparent solution, indicating the conversion of **9** into more soluble D−A adducts. Finally, a crude mixture of different adducts was separated and purified by recycling gel permeation chromatography (GPC) to afford PP **3** in 30% yield over two steps.

The concept underlying the synthesis of the even larger dendritic PP **4** with 546 carbons is the radial layer‐by‐layer extension from a HPB core (Scheme 2). The inner oligophenylene layer (indicated in blue in Scheme 2) can be installed onto HPB **13** ^[25]^ by reaction with 3‐*m*‐(triisopropylsilylethynyl)phenyl‐2,4,5‐triphenylcyclopenta‐2,4‐dien‐1‐one (**12**). The protected ethynyl group of **12** enables the further radial extension. The outer layer (indicated in red in Scheme 2) contains 24 more benzene units than the inner one, and thus 2,5‐bis(3,4‐diphenyl)phenyl‐3,4‐diphenylcyclopenta‐2,4‐dien‐1‐one (**18**), a CP derivative with four more phenyl substituents, was selected for constructing this layer (Scheme 2).

**Scheme 2:**
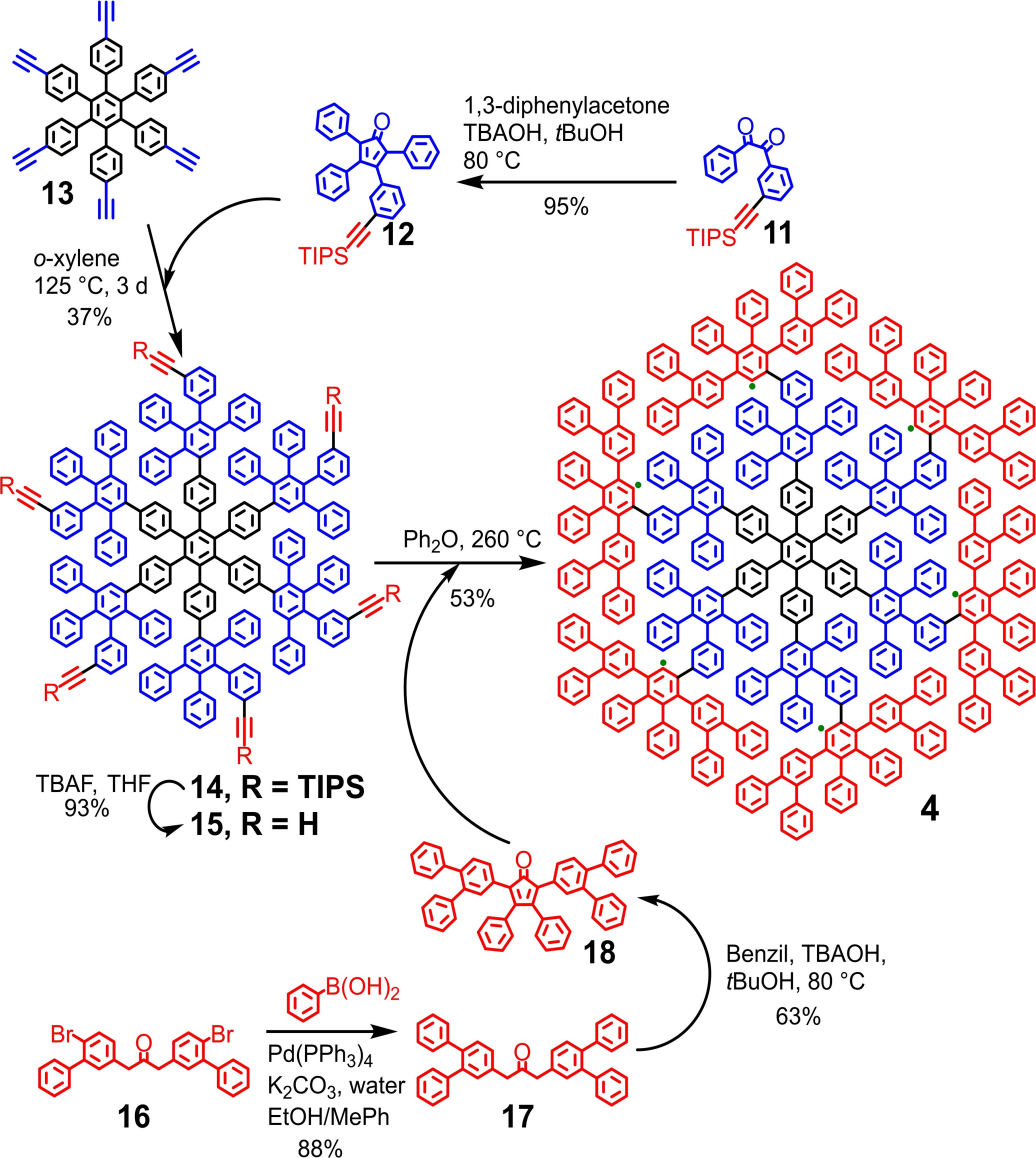
Synthesis of dendritic PP **4**. TBAOH: tetrabutylammonium hydroxide.

CP **12** was prepared by a twofold aldol condensation between 3‐(triisopropylsilylethynyl)benzil (**11**)^[26]^ and 1,3‐diphenylacetone in 95% yield. In parallel, synthesis of CP **18** was conducted by firstly introducing two additional phenyl groups onto 1,3‐di[(4‐bromo‐3‐phenyl)phenyl]acetone (**16**)^[27]^ via a twofold Suzuki reaction, followed by an aldol condensation with benzil (Scheme 2). With the CPs **12** and **18** in hand, HPB **13** ^[25]^ equipped with six ethynyl groups was radially extended by a six‐fold D−A reaction with CP **12** (Scheme 2). After removing the triisopropylsilyl protecting groups, dendritic PP **15** was isolated as an ethynyl‐functionalized derivative of PP **2**. Dendritic PP **15** was then further radially extended to construct the outer oligophenylene layer by a six‐fold D−A reaction with CP **18** in diphenyl ether at 260 °C, leading to dendritic PP **4** in 53% yield.

Both dendritic PPs **3** and **4** are soluble in common organic solvents such as THF, dichloromethane, toluene, and chloroform, thus allowing their characterization by solution NMR. Unfortunately, the ^1^H NMR spectra of both PPs **3** and **4** (Figures S4 and S5) are complex due to extensive signal overlap, which hinders unambiguous structural analyses. Nevertheless, if assigning the signal at the lowest field in the ^1^H NMR spectra of PPs **3** and **4** to the signal of the correspondingly most deshielded protons (marked as green in Scheme 1 and Scheme 2), their integration compared with that of the remaining protons is in good agreement with the theoretical ratio in both PPs **3** and **4** (Figure S4 and Figure S5).

The six‐fold reactions applied during the growth of both dendritic PPs **3** and **4** can potentially lead to the formation of lower‐molecular‐weight byproducts when reaction fails at one of the six arms. However, no signal was observed at around 3 ppm in the ^1^H NMR spectra nor at 50–100 ppm in the ^13^C NMR spectra of both PPs **3** and **4** (Figure S4 and Figure S5). This suggests the absence of sp‐hybridized carbons in the skeleton of both PPs **3** and **4**, indicating the completion of the D−A reaction steps. Furthermore, MALDI‐TOF spectra of **3** and **4** show distinct peaks at *m/z*=4639.9 and 6920.8, respectively, in agreement with their exact mass (Figure 2). The isotopic distribution for both **3** and **4** matches their theoretical patterns (Figure 2, insets), further corroborating the formation of these dendritic PPs. To estimate the particle size of dendritic PPs **3** and **4** in solution, we performed their ^1^H NMR DOSY measurements (see supporting information for details). Importantly, if there exists lower‐molecular‐weight byproduct, proton signals are expected to appear at different hydrodynamic radii (*R*_H_). However, the aromatic proton signals in the ^1^H NMR spectra of dendritic PPs **3** and **4** all belong to molecular species with *R*_H_ of 1.6±0.1 and 2.0±0.1 nm, respectively, in CD_2_Cl_2_ solutions, supporting the purity of these samples. The *R*_H_ of HPB **1** and PP **2** were determined to be 0.62±0.03 and 1.3±0.1 nm, respectively, by the same method, validating the extended molecular sized of PPs **3** and **4**.

**Figure 2:**
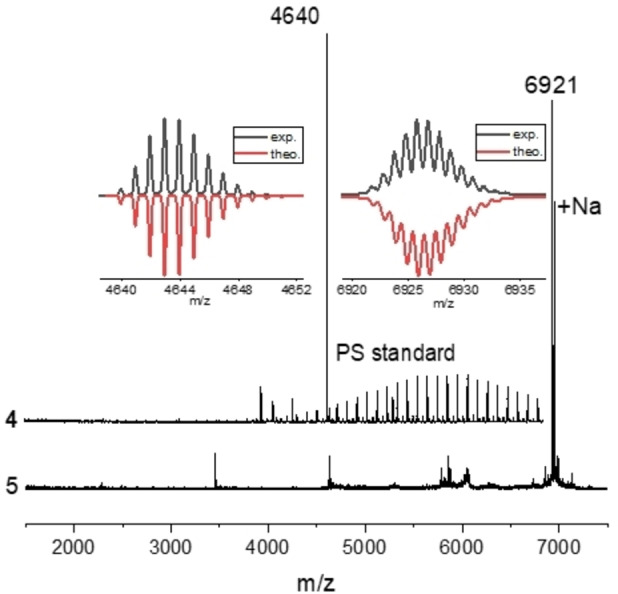
MALDI‐TOF mass spectra of **3** (upper) and **4** (lower). Insets: comparison of isotopes distribution and the corresponding theoretical patterns.

The synthesis of PP **3** leads to a single regioisomer. In contrast, PP **4** can be a mixture of isomers since the D−A reaction between CP **12** and HPB **13** is not regioselective (Figure S1). This reflects itself in broader ^1^H NMR signals for PP **4** in comparison to those of PP **3** (Figure S4 and S5). Nevertheless, all the regioisomers of **4** can be represented as 2D projections without spatially overlapping benzene rings (Figure S1), and can be potentially converted to a single hexagonal NG after cyclodehydrogenation reactions. After obtaining the giant PPs **3** and **4**, we investigated their cyclodehydrogenation reactions under various conditions,[^28^, ^29^] such as Cu(CF_3_SO_3_)_2_/AlCl_3_, FeCl_3_, 2,3‐dichloro‐5,6‐dicyano‐*p*‐quinone/CF_3_SO_3_H, and Cu(CF_3_SO_3_)_2_/AlCl_3_/h*ν*. Unfortunately, all attempts led to insoluble product mixtures without indication of the complete cyclodehydrogenation to the corresponding NGs based on the MALDI‐TOF MS analyses (see Figure S21 for a representative result). This could be due to steric hindrance and possibly formed stable radical cation species,^[30]^ being reminiscent of previous observations during oxidative cyclodehydrogenation of dendritic PPs larger than PP **2**.^[19]^ Notably, solution‐based synthesis of large NGs with up to 258 sp^2^ carbons has been recently reported,^[31]^ albeit non‐planarity is implanted into the molecular structure.[^32^, ^33^, ^34^] This can be an alternative approach to overcome the size limit of the current NG synthesis, while the synthesis of giant planar NGs remains a distinct challenge.

The on‐surface cyclodehydrogenation has recently emerged as an efficient alternative approach to synthesize NGs that cannot be obtained in solution because of stability and/or solubility issues.[^21^, ^22^, ^23^] Importantly, the corresponding precursor is generally deposited onto metal substrates under UHV conditions by sublimation, which cannot be achieved for high‐molecular‐weight precursors like large dendritic PPs. To this end, HV‐ESD allows deposition of macromolecules onto substrates without the need of thermal evaporation.[^35^, ^36^, ^37^, ^38^, ^39^, ^40^] Thus, we attempted the deposition of both dendritic PPs **3** and **4** onto Au(111) surface by the HV‐ESD technique (see supporting information for details). Figure 3a exhibits an nc‐AFM image of dendritic PP **3** deposited on Au(111) at room temperature. Notably, bright protrusions are observed with a 6 nm size, which is reasonably close to that of dendritic PP **3** (Figure 3ab). This observation indicates that the PP **3** molecules have been successfully deposited on the Au(111) surface. The height profile of these objects displays an uneven topology with a peak height around 7 Å (Figure 3b,d), in agreement with the 3D structure of PP **3**.

**Figure 3:**
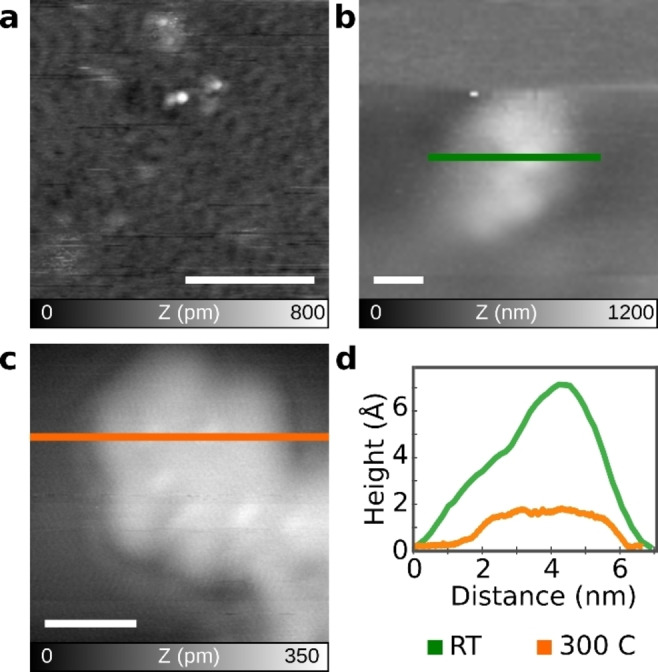
nc‐AFM images of dendritic PP **3** on Au(111) surface under UHV conditions (a)(b) before and (c) after annealing at 300 °C and (d) topography profiles extracted from (b)(c). Parameters: (a) f_2_=989 kHz, A_2_=600 pm, Δf=−10 Hz; (b) f_1_=159 kHz, A_1_=2 nm, Δf=−20 Hz; (c) f_2_=988 kHz, A_2_=800 pm, Δf=−60 Hz. Scale bar: (a) 50 nm, (b)(c) 2 nm.

Next, the sample was annealed at 300 °C for 20 min to attempt the surface‐assisted cyclodehydrogenation of dendritic PP **3**. As demonstrated in an enlarged image of an isolated single molecule (Figure 3c), a quasi‐hexagonal object was observed with a height of around 2 Å (Figure 3d), which was significantly smaller than those measured before annealing. Although the acquisition of atomically‐resolved images was challenging, precluding the elucidation of the resulting chemical structures, this height is in good agreement to the height of other graphene‐like molecules measured by nc‐AFM.[^41^, ^42^] These observations suggest the occurrence of thermally induced cyclodehydrogenations at least to some extent. Similar structural features are also noticed for dendritic PP **4** after annealing (Figure S3). The results point to the potential of utilizing on‐surface cyclodehydrogenation for the synthesis of giant NGs, although convincing evidence for a complete planarization could not be obtained in this work.

In summary, we developed a “layer‐by‐layer” radial extension strategy to synthesize giant dendritic PPs with up to 546 carbons. These dendritic PPs are suitable precursors for unprecedentedly large NGs with diameters approaching 5 nm. Whereas their cyclodehydrogenations in solution failed, HV‐ESD allowed to deposit these giant molecules onto metal surfaces. Further thermally assisted cyclodehydrogenation of both dendritic PPs on surface largely flattened their structure, showing the potential of on‐surface graphitization of large NG precursors. These results describe the scope and limitations of the two‐step NG synthesis when applied to larger and larger hexagons, and provides insights for further pushing the size limits of bottom‐up NG synthesis.

## Conflict of interest

The authors declare no conflict of interest.

## Supporting information

As a service to our authors and readers, this journal provides supporting information supplied by the authors. Such materials are peer reviewed and may be re‐organized for online delivery, but are not copy‐edited or typeset. Technical support issues arising from supporting information (other than missing files) should be addressed to the authors.

Supporting Information

## Data Availability

The data that support the findings of this study are available in the supplementary material of this article.
