# Supplementary material for: Synthesis of Giant Dendritic Polyphenylenes with 366 and 546 Carbon Atoms and Their High‐vacuum Electrospray Deposition
Source: Chem Asian J. 2022 Apr 22;17(11):e202200220. doi: 10.1002/asia.202200220 (PMC9321752; doi:10.1002/asia.202200220)
Supplement: Supplementary file 1 — Supporting Information [file ASIA-17-0-s001.pdf]

CHEMISTRY
AN ASIAN JOURNAL

Supporting Information

Synthesis of Giant Dendritic Polyphenylenes with 366 and 546 Carbon Atoms and Their High-vacuum Electrospray Deposition

Ian Cheng-Yi Hou[+], Antoine Hinaut[+], Sebastian Scherb, Ernst Meyer,* Akimitsu Narita,* and Klaus Müllen*© 2022 The Authors. Chemistry - An Asian Journal published by Wiley-VCH GmbH. This is an open access article under the terms of the Creative Commons Attribution License, which permits use, distribution and reproduction in any medium, provided the original work is properly cited.

1. General Information

Unless otherwise noted, materials were purchased from Fluka, Aldrich, Acros, abcr, Merck, and other commercial suppliers, and were used as received. Au substrates were purchased from Mateck GmbH. Compounds **5**[1], **8**[2], **11**[3], **13**[2], and **16**[4] were synthesized according to reported procedures. All reactions working with air- or moisture-sensitive compounds were carried out under argon atmosphere using standard Schlenk line techniques. Thin layer chromatography (TLC) was performed on silica gel-coated aluminium sheets with F254 indicator. Preparative column chromatography was performed on silica gel from Merck with a grain size of 0.063–0.200 mm or 0.04–0.063 mm (flash silica gel, Geduran Si 60). Melting points were determined on a Büchi hot stage apparatus without correction. Membrane filtration was performed on polyvinylidene fluoride membranes with a pore size of 0.45 μm (Merck). NMR spectra were recorded in deuterated solvents using Bruker AVANCE III 300 and Bruker AVANCE III 700 MHz NMR spectrometers. Chemical shifts (δ) were expressed in ppm relative to the residual of solvent (CD_2Cl_2 @ 5.32 ppm for ^1H NMR, 53.84 ppm for ^{13}C NMR; dimethylsulfoxide-d_6 (DMSO-d_6) @ 2.50 ppm for ^1H NMR, 39.52 ppm for ^{13}C NMR). Coupling constants (J) were recorded in Hertz (Hz) with multiplicities explained by the following abbreviations: s = singlet, d = doublet, t = triplet, q =quartet, dd = doublet of doublets, dt = doublet of triplets, m = multiplet, br = broad. The ^{13}C NMR spectra of compounds **3** and **4** were recorded with spin-echo attached-proton test sequence (APT) with CH, CH_3 showing negative signal and C, CH_2 showing positive signal. High-resolution mass spectra (HRMS) were recorded by matrix-assisted laser decomposition/ionization time of flight (MALDI-TOF) mass spectrometry with a Bruker Reflex II-TOF spectrometer (MALDI-TOF HRMS) in reflection mode. Except for compound **9** and the cyclodehydrogenated products, where 7,7,8,8-tetracyanoquinodimethane (TCNQ) was used as matrix, all the rest of the compounds were measured using *trans*-2-[3-(4-*tert*-butylphenyl)-2-methyl-2-propenylidene]malononitrile (DCTB) as matrix. Non-contact atomic force microscopy (nc-AFM) measurements were performed with a home-built microscope with Nanonis RC5 electronics. PPP-NCL cantilevers (Nanosensors) were used as sensors (typical resonance frequencies of f1 = 160 kHz and f2 = 1 MHz).

2. Experimental details

Isomers of dendritic PP 4

The Diels-Alder (D-A) reaction between 2,3,4,5-tetraphenylcyclopenta-2,4-dien-1-one (CP) and ethynyl group is not regioselective.[5] Accordingly, the six-fold D-A reaction between CP **12** and PP **13** can lead to two different connections on each "side arm" in dendritic PP **14** (Figure S1). Thus, PP **14** can be a mixture of six types of isomer: A_6, A_5B, A_4B_2 (three regioisomers), A_3B_3 (three regioisomers), A_2B_4 (three regioisomers), AB_5, and B_6. If every isomer exists, then dendritic PP **14** is a mixture of 13 different regioisomers in total. Since the local environments are similar around the ethynylene groups in each isomer, after removing the triisopropylsilyl protecting groups, the resulting ethynyl groups shall possess the same reactivity toward the D-A reaction with CP **18** (without formation of additional isomer). Thus, PP **4** is also likely a mixture of 13 isomers. This scenario can also complicate the cyclodehydrogenation reactions of PP **4**.

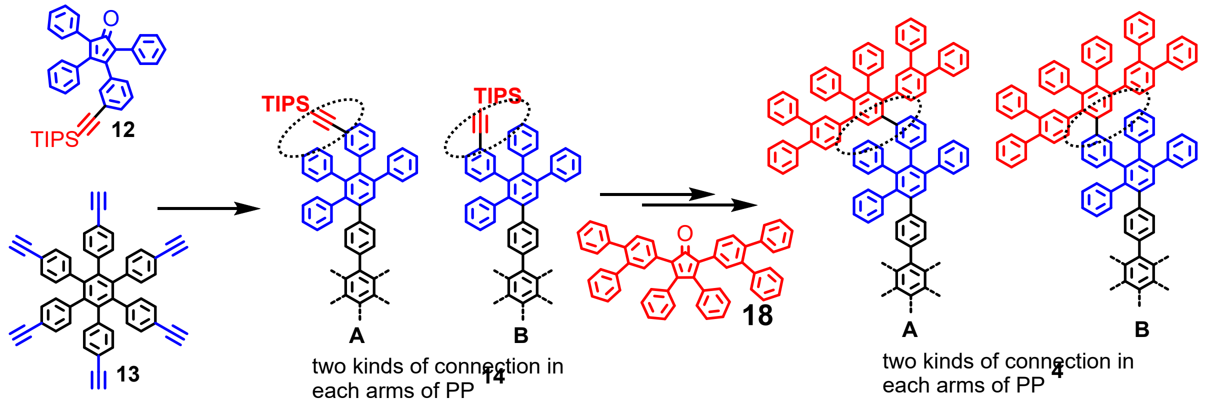

Figure S1. The two kinds of connection in each arm of PP **14** formed after D-A reaction between CP **12** and PP **13**.

^{1}H NMR diffusion ordered spectroscopy (DOSY) analysis

The particle size of PPs **1**–**4** in solution was estimated by means of their ^{1}H NMR DOSY spectra (Figure S4). Van der Waals radius was estimated using the Stokes-Einstein equation:

$$r = \frac{kT}{6\pi\eta D}$$

where k is Boltzmann constant (1.380 × 10^{-23} J/K), T is temperature in Kelvin, η is viscosity of solution (CD_2Cl_2, 4.17 × 10^{-4} Pa·s at 25 °C) and D is the self-diffusion constant extracted from ^{1}H NMR DOSY spectra (m^2/s). The diffusion constants were calibrated by CH_2Cl_2 in CD_2Cl_2 solvent (D = 3.03 × 10^{-5} cm^2/s) as internal standard.

The extracted information is summarized in Table S1, assuming that the PP molecules have spherical topologies in solution. The hydrodynamic radii increase from HPB **1** to PP **4**, in agreement with the increasing molecular weight. The intraparticle density (ρ_P) of HPB **1** in solution, defined as its mass divided by its hydrodynamic volume, is determined as 0.9 ± 0.1 g/cm^3 (Table S1). The value is close to the density of benzene (0.876 g/cm^3 at 20 °C), reflecting the twisted and tightly interlocked nature of the phenyl groups. In contrary, the ρ_P of the larger dendritic PPs **2**–**4** quickly drop with the increasing molecular weight, indicating large intramolecular free volume occupied by solvent molecules. Notably, the largest PP **4** possesses a different "backbone" from those of PPs **1**–**3**; namely, backbones of the latter three are composed of only rigid *p*-phenylenes while that of **4** contains more flexible *m*-phenylene substructures. This could affect their intramolecular packing density and thus intramolecular free volume. However, the ρ_P of PP **4** is significantly smaller than that of PP **3**, suggesting that the side arms do not backfold onto the HPB core to fill the intramolecular free volume. This is in relationship with the semi-rigid[5] and "surface dense"[6] nature of PP dendrimers that would be helpful for "preprogramming" the dendritic PPs into a conformation that is suitable for further cyclodehydrogenation.

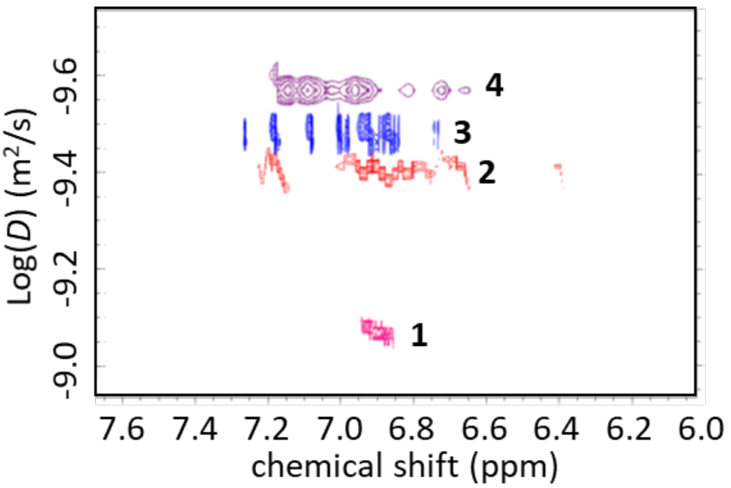

Figure S2. ^{1}H NMR DOSY signal of dendritic PPs **1**–**4** in CD_2Cl_2 at 25 °C.

Table S1. Molecular weight (M), self-diffusion constants (D), hydrodynamic radii (R_H), hydrodynamic volume (V_H), and intraparticle density (ρ_P) of dendritic PPs **1**, **2**, **3**, and **4** particles in CD_2Cl_2, estimated by ^1H NMR DOSY spectra at 25 °C.

Dendritic PPs	M (kg/mol)	D[a] (10^{-6} cm^2/s)	R_H[b] (nm)	V_H[c] (nm^3)	ρ_P[d] (g/cm^3)
**1**	0.535	8.4 ± 0.4	0.62 ± 0.03	1.0 ± 0.1	0.9 ± 0.1
**2**	2.82	3.9 ± 0.2	1.3 ± 0.1	10 ± 2	0.47 ±0.01
**3**	4.64	3.2 ± 0.2	1.6 ± 0.1	18 ± 3	0.43 ± 0.06
**4**	6.93	2.7 ± 0.1	2.0 ± 0.1	32 ± 4	0.36 ± 0.05

[a] Directly extracted from DOSY NMR spectra. [b] Calculated from D applying Stokes-Einstein equation. [c] Calculated from $4/3 \times \pi \times R_H^3$. [d] Calculated from M/V_H.

High vacuum electrospray deposition (HV-ESD) on Au(111) surface and nc-AFM measurements

Au(111) single crystal substrates were prepared under ultra-high vacuum (UHV) conditions by several cycles of Ar^+ sputtering and annealing at 550 °C. After the treatment, atomically flat surfaces were obtained with large terraces separated by atomic steps. The HV-ESD was performed on Au(111) samples kept at a room temperature following our previous procedure[7] using a commercial system (MolecularSpray), with a typical applied voltage of 1.5 kV. The setup was connected to preparation chambers of a UHV system. Dendritic PPs **3** and **4** were deposited for 10 min from toluene/methanol solution (five to one, ratio in volume). During the HV-ESD, pressure of vacuum chamber rose up to 1×10^{-6} mbar. Samples were first scanned prior to any annealing process. The surface-assisted thermal cyclodehydrogenation were performed by *in-situ* annealing for 20 min at 300 °C under UHV. Prior to the measurements, scanning tips were annealed for 1 h at 100 °C followed by Ar^+ sputtering for 90 s at 680 eV at an Ar^+ pressure of 3×10^{-6} bar. Base pressure of the UHV system was maintained at 2×10^{-11} mbar during measurements. nc-AFM imaging was performed at room temperature on our home-built microscope. Either first or second resonance were used.

*Dendritic PP **4** on Au(111) after annealing at 300°C:*

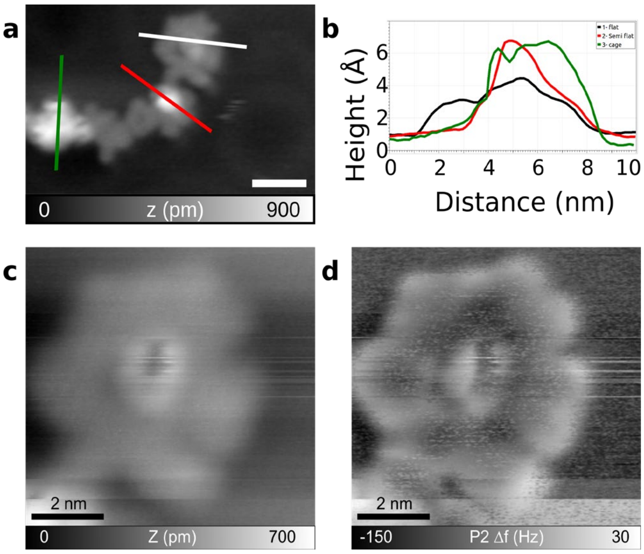

Figure S3. nc-AFM images of dendritic PP **4** on Au(111) surface under UHV conditions after annealing at 300 °C. (a) Large scale image and (b) corresponding profile of different molecules shown in the same color code. (c,d) Multipass nc-AFM image of a single molecule. Parameters: f_2 = 1.01 MHz, A_2 = 400 pm; (a) $\Delta f_2 = -20$ Hz, (c) $\Delta f_2 = -30$ Hz; (d) multipass from (c) with $\Delta z = -200$ pm. Scale bar: (a) 5 nm, (c,d) 2 nm.

Synthetic procedure and characterization data for new compounds

*Synthesis of dendritic PP **3***: A mixture of star-shape skeleton **9** (30 mg, 0.012 mmol), 2,3,4,5-tetraphenyl-cyclopenta-2,4-dien-1-one (46 mg, 0.12 mmol) and diphenyl ether (0.5 mL) was degassed by freeze-pump-thaw technique for three cycles. This mixture was heated at 260 °C overnight, during which it became a clear solution, and the purple color lightened. After cooling to a room temperature, methanol was added to the solution. A white precipitate (47 mg, 0.010 mmol, 83%) was collected. This precipitate was further purified by recycling gel permeation chromatography to yield **3** as a white solid (37 mg, 0.079 mmol, 66%): mp > 410 °C; ^{1}H NMR (700 MHz, CD_2Cl_2): δ 7.22 (s, 6H), 7.17–7.12 (m, 36H), 7.08– 7.02 (m, 24H), 6.99–6.93 (m, 36H), 6.93–6.86 (m, 84H), 6.86–6.79 (m, 48H), 6.70 (d, J = 8 Hz, 12H); ^{13}C NMR (176 MHz, CD_2Cl_2) δ 141.49, 141.34, 141.21, 141.18, 141.07, 140.90, 140.84, 140.41, 140.39, 140.33, 140.24, 140.04, 139.91, 137.98, 137.57, 132.46, 132.17, 132.13, 131.88, 131.84, 131.81, 130.38, 128.83, 127.35, 127.19, 127.05, 126.98, 126.94, 125.85, 125.70 125.63, 125.49, 125.26, 123.36; MS (MALDI-TOF) m/z (%): $[M]^+$ calcd for $C_{366}H_{246}$ 4639.9250; Found 4639.9245.

*Synthesis of dendritic PP **4**:* A solution of dendritic PP **14** (55 mg, 0.014 mmol) in tetrahydrofuran (THF) (3 mL) was degassed by freeze-pump-thaw technique for three cycles. To this solution was injected a THF solution of tetrabutylammonium fluoride (TBAF) (0.1 mL, 0.09 mmol, 1 M). The solution was allowed to react for 1 h, and poured into methanol (30 mL) to induce precipitation of the product. The precipitate was collected by filtration with a membrane filter, and washed with methanol to give dendritic PP **15** as a white powder (38 mg, 0.013 mmol, 93% crude yield), which was directly used in the next step without further purification. A solution of **15** (35 mg, 0.012 mmol) and 2,5-di([1,1':2',1''-terphenyl]-4'-yl)-3,4-diphenylcyclopenta-2,4-dien-1-one (**18**) (73 mg, 0.11 mmol) in diphenyl ether (1 mL) was degassed by freeze-pump-thaw technique for three cycles. This mixture was heated at 260 °C overnight. After cooling to a room temperature, methanol (10 mL) was added to the

solution to induce precipitation of product. The precipitate was collected by filtration with a membrane filter, and washed with methanol to give crude **4** as a white powder (62 mg, 0.0090 mmol, 75%). The crude **4** was further purified by recycling gel permeation chromatography to yield **4** as a white solid (44 mg, 0.0064 mmol, 53%): mp > 410 °C; ^{1}H NMR (700 MHz, CD_2Cl_2): δ 7.56–7.36 (br, 6H), 7.33–6.48 (m, 348H), 6.48–6.29 (m, 12H); ^{13}C NMR (176 MHz, CD_2Cl_2): δ 142.36, 142.30, 142.25, 142.11, 141.96, 141.90, 141.80, 141.65, 141.02, 140.97, 140.84, 140.70, 140.60, 140.49, 140.44, 140.39, 140.29, 140.23, 140.04, 139.49, 139.45, 139.32, 138.95, 138.79, 138.01, 134.58, 133.64, 132.93, 132.31, 132.18, 132.12, 132.05, 132.02, 131.78, 131.72, 131.58, 131.54, 130.25, 130.24, 130.17, 129.62, 129.43, 128.73, 128.19, 128.11, 128.06, 127.91, 127.52, 127.23, 126.97, 126.77, 126.59, 126.14, 125.93, 125.84; HRMS (MALDI-TOF) *m/z*: $[M]^+$ calcd for $C_{546}H_{366}$ 6920.9 (2), 6921.9 (8), 6922.9 (26), 6923.9 (55), 6924.9 (84), 6925.9 (100), 6926.9 (98), 6927.9 (82), 6928.9 (59), 6929.9 (37), 6930.9 (20), 6931.9 (8), 6932.9 (2); Found 6920.8 (4), 6921.7 (13), 6922.7 (30), 6923.7 (57), 6924.7 (81), 6925.7 (100), 6926.8 (98), 6927.7 (83), 6928.7 (61), 6929.7 (39), 6930.7 (25), 6931.7 (13), 6932.7 (8).[8]

*Synthesis of 1-(4-bromophenylethynyl)-3,5-diphenylbenzene (**6**)*: To a solution of 1-(trimethylsilylethynyl)-3,5-diphenylbenzene (**5**)[1] (5.07 g, 15.5 mmol) in THF (50 mL) was injected a THF solution of TBAF (23 mL, 1 M, 23 mmol). The solution was stirred at a room temperature for 1 h, and then passed through a short pad of silica gel with THF as eluent. Solvent was then removed *in vacuo* to afford 1-ethynyl-3,5-diphenylbenzene as a white solid (3.92 g, 15.4 mmol, 99% crude yield), which was directly used in the next step without further purification. A mixture of 1-ethynyl-3,5-diphenylbenzene (3.92 g, 15.4 mmol), $PdCl_2(PPh_3)_2$ (0.22 g, 0.31 mmol), CuI (0.059 g, 0.31 mmol), THF (20 mL) and triethylamine (10 mL) was degassed by argon bubbling under vigorous stirring for 15 min. To this mixture was then added *p*-bromoiodobenzene (4.81 g, 17.0 mmol) at 0 °C, portion by portion. The mixture was gradually warmed up to a room temperature and stirred overnight. Diethyl ether and ice were added into the mixture and the organic layer was washed with HCl (aq., 2 M), water, and brine, and dried over $MgSO_4$. The solution was concentrated *in vacuo* and the residue was purified by recrystallization from ethanol to afford **6** as a pale yellow crystal (3.21 g, 7.86 mmol, 51%, over two steps): mp 121.9–123.2 °C; ^{1}H NMR (300 MHz, CD_2Cl_2): δ 7.83 (d, J = 1.7 Hz, 1H), 7.77 (d, J = 1.7 Hz, 2H), 7.69 (d, J = 7.3 Hz, 4H), 7.54 (d, J = 8.4 Hz, 2H), 7.50 (t, J = 7.3 Hz, 4H), 7.46 (d, J = 8.4 Hz, 2H), 7.41 (t, J = 7.3 Hz, 2H); ^{13}C NMR (75 MHz, CD_2Cl_2): δ 142.62, 140.72, 133.67, 132.28, 129.57, 129.48, 128.41, 127.71, 126.88, 124.38, 123.16, 122.71, 90.92, 88.94; MS (MALDI-TOF) *m/z* (%): $[M+H_2]^+$ calcd for $C_{26}H_{19}Br$ 410.1 (99), 411.1 (28), 412.1 (100), 413.1 (27), 414.1 (4); Found 409.7 (100), 410.7 (61), 411.7 (100), 412.7 (61), 413.7 (8).

Synthesis of {4-([1,1':3',1''-terphen]-5'-ylethynyl)phenyl}boronic acid (7): To a solution of 1-(4-bromophenylethynyl)-3,5-diphenylbenzene (**6**) (3.11 g, 7.62 mmol) in THF (25 mL) was injected a hexane solution of *n*-butyllithium (5.3 mL, 8.4 mmol) at –78 °C. The mixture was allowed to react for 30 min, and then $B(OMe)_3$ (1.7 mL, 15 mmol) was added via syringe. The solution was then gradually warmed up to a room temperature, and further stirred for 3 h. An aqueous solution of HCl (20 mL, 2 M) was added at 0 °C to quench the reaction. The mixture was then warmed up to a room temperature and vigorously stirred for 4 h. Diethyl ether (200 mL) was added and the organic phase was washed with water and brine, and dried over $MgSO_4$. Solvent was then removed *in vacuo* and the residue was purified by recrystallization from $CHCl_3$ to afford compound **7** as a white powder (1.89 g , 5.05 mmol, 66%): mp 227.7–230.6 °C; ^{1}H NMR (300 MHz, DMSO-d_6): δ 8.20 (s, 2H), 7.94 (s, 1H), 7.89 – 7.77 (m, 8H), 7.58 (d, J = 7.8 Hz, 2H), 7.51 (t, J = 7.4 Hz, 4H), 7.42 (t, J = 7.4 Hz, 2H); ^{13}C NMR (75 MHz, DMSO- d_6): δ 141.54, 139.16, 134.38, 130.46, 129.09, 128.54, 128.10, 127.13, 125.76, 123.70, 123.65, 94.78, 90.18, 90.02; HRMS (MALDI-TOF) *m/z*: $[M]^+$ calcd for $C_{26}H_{19}BO_2$ 374.1478; Found 374.1471.

*Synthesis of star-shape skeleton **9**:* A mixture of {4-([1,1':3',1''-terphen]-5'-ylethynyl)phenyl}boronic acid (**7**) (0.26 g, 0.70 mmol), hexa(*p*-iodophenyl)benzene (**8**)[2] (90 mg, 0.069 mmol), and K_2CO_3 (1.2 g, 8.7 mmol) in water (4.5 mL), EtOH (1.5 mL), and toluene (20 mL) was degassed by freeze-pump-thaw technique for one cycle. $Pd(PPh_3)_4$ (4 mg, 0.003 mmol) was then added, and the mixture was further degassed by freeze-pump-thaw technique for two cycles. The mixture was then heated at 80 °C under vigorously stirring for two days. After cooling to a room temperature, the mixture was poured into methanol. A white solid was collected as crude **9** (79 mg, 0.031 mmol, 45% crude yield). This solid was used for the next step without further purification: mp > 410 °C; NMR spectra could not be recorded because of a very low solubility in common organic solvents; HRMS (MALDI-TOF) *m/z*: $[M]^+$ calcd for $C_{198}H_{12}$ 2502.9860; found 2502.9988.[8]

Synthesis of 2,3,5-triphenyl-4-{3-[(triisopropylsilyl)ethynyl]phenyl}cyclopenta-2,4-dien-1-one (12): To a *t*BuOH (300 mL) solution of 1,3-diphenylacetone (1.13 g, 5.37 mmol) and 3-[(triisopropylsilyl)ethynyl]benzil (**11**)[3] (2.14 g, 5.48 mmol) was added at 80 °C tetrabutylammonium hydroxide (TBAOH) (0.71 g, 1.1 mmol, 40% w/w in EtOH, two portions). The solution turned dark purple immediately. The reaction was monitored by thin layer chromatography until starting material was consumed (about 30 min). After cooling to a room temperature, diethyl ether was added, and the organic phase was washed with NH_4Cl (aq., sat.), water, and brine, and dried over $MgSO_4$. Solvent was removed *in vacuo* and residue was purified by silica gel column chromatography (dichloromethane/hexane = 1/6) to yield **12** as a purple solid (2.9 g, 5.1 mmol, 95%): mp 75.6–77.7 °C; ^{1}H NMR (300 MHz, CD_2Cl_2): δ 7.41–7.17 (m, 14H), 7.13 (t, J = 7.8 Hz, 1H), 7.02–6.88 (m, 4H), 1.07 (s, 21H); ^{13}C NMR (75 MHz, CD_2Cl_2): δ 200.72, 155.22, 154.05, 133.76, 133.63, 133.60, 132.11, 131.43, 131.25, 130.67, 129.89, 129.75, 129.20, 128.61, 128.61, 128.53, 128.25, 128.06, 126.41, 125.98, 123.72, 118.74, 106.86, 91.86, 18.94, 11.81; MS (MALDI-TOF) *m/z* (%): $[M]^+$ calcd for $C_{40}H_{40}OSi$ 564.3 (100), 565.3 (48), 566.3 (15); Found 564.3 (100), 565.3 (51), 566.3 (14).

*Synthesis of dendritic PP **14**:* A mixture of hexa(4-ethynylphenyl)benzene (**13**)[2] (30 mg, 0.044 mmol), 2,3,5-triphenyl-4-{3-[(triisopropylsilyl)ethynyl]phenyl}cyclopenta-2,4-dien-1-one (**12**) (226 mg, 0.398 mmol), and *o*-xylene (0.6 mL) was degassed by freeze-pump-thaw technique for three cycles. This mixture was heated at 125 °C for three days. After cooling to a room temperature methanol (3 mL) was added to induce precipitation of product. The precipitate was collected by filtration with a membrane filter and washed with methanol to give **13** as a white powder (61 mg, 0.015 mmol, 37%): mp > 410 °C; ^{1}H NMR (300 MHz, CD_2Cl_2): δ 7.45–7.37 (br, 6H), 7.26–7.08 (m, 30H), 7.06–6.68 (m, 84H), 6.68–6.57 (br, 12H), 6.43–6.30 (m, 12H), 1.08 (s, 126H); ^{13}C NMR (75 MHz, CD_2Cl_2): δ 142.16, 142.11, 141.97, 141.20, 141.03, 140.74, 140.64, 140.38, 140.20, 139.51, 139.38, 138.97, 138.54, 135.98, 131.99, 131.59, 130.31, 128.68, 128.02, 127.91, 127.74, 127.28, 127.16, 127.01, 126.74, 126.14, 125.88, 122.38, 122.08, 107.58, 107.51, 90.34, 90.22, 18.83, 18.81, 11.70; MS (MALDI-TOF) *m/z* (%): $[M\text{-}CH]^+$ calcd for $C_{287}H_{269}Si_6$ 3883.0 (14), 3884.0 (48), 3885.0 (84), 3886.0 (100), 3887.0 (92), 3888.0 (68), 3889.0 (43), 3890.0 (22), 3891.0 (7); Found 3883.3 (12), 3884.3 (44), 3885.3 (82), 3886.3 (100), 3887.3 (94), 3888.3 (71), 3889.3 (47), 3890.3 (25), 3891.3 (13).[8]

Synthesis of 1,3-di([1,1':2',1''-terphenyl]-4'-yl)propan-2-one (17): The procedure was modified from our previous work describing synthesis of a similar compound.[4] A mixture of 1,3-bis(3-phenyl-4-bromophenyl)acetone (**16**)[4] (113 mg, 0.218 mmol), phenyl boronic acid (80 mg, 0.65 mmol), and K_2CO_3 (180 mg, 1.3 mmol) in water (3 mL), EtOH (3 mL), and toluene (12 mL) was degassed by freeze-pump-thaw technique for one cycle. $Pd(PPh_3)_4$ (25 mg, 0.022 mmol) was then added, and the mixture was further degassed by freeze-pump-thaw technique for two cycles. The mixture was then heated at reflux

under vigorously stirring for 4 h. After cooling to a room temperature, the mixture was diluted with diethyl ether and washed with NaOH (aq., 2 M), water, and brine, and dried over $MgSO_4$. Solvent was then removed *in vacuo*, and the residue was purified by silica gel column chromatography (dichloromethane/hexane = 2/3) to yield **17** as a white solid (99 mg, 0.19 mmol, 88%): mp 138.8–140.2 °C; ^{1}H NMR (300 MHz, CD_2Cl_2): δ 7.39 (d, J = 7.8 Hz, 2H), 7.34–7.17 (m, 16 H), 7.17–7.06 (m, 8 H), 3.91 (s, 4 H); ^{13}C NMR (75 MHz, CD_2Cl_2): δ 205.74, 141.88, 141.78, 141.34, 139.86, 134.05, 132.39, 131.41, 130.42, 130.39, 129.27, 128.39, 127.10, 127.02, 118.73, 49.44; MS (MALDI-TOF) *m/z* (%): $[M]^+$ calcd for $C_{39}H_{30}O$ 514.2 (100), 515.2 (42), 516.2 (9); Found 514.2 (100), 515.2 (48), 516.2 (9).

*Synthesis of 2,5-di([1,1':2',1''-terphenyl]-4'-yl)-3,4-diphenylcyclopenta-2,4-dien-1-one (**18**):* A solution of 1,3-di([1,1':2',1''-terphenyl]-4'-yl)propan-2-one (**17**) (1.01 g, 1.96 mmol) and benzil (412 mg, 1.96 mmol) in *t*BuOH (150 mL) was heated to 80 °C, and then TBAOH (0.19 mg, 0.29 mmol, 40% w/w in EtOH) was added. The solution turned dark purple immediately. The reaction was monitored by thin layer chromatography until consumption of starting material (about 30 min). After cooling to a room temperature, diethyl ether was added, and the organic phase was washed with NH_4Cl (aq., sat.), water, and brine, and dried over $MgSO_4$. Solvent was removed *in vacuo*, and the residue was purified by silica gel column chromatography (dichloromethane/hexane = 1/6) to yield **18** as a purple solid (0.88 g, 1.2 mmol, 63%): mp 143.8–145.0 °C; ^{1}H NMR (300 MHz, CD_2Cl_2): δ 7.50–7.25 (m, 12H), 7.25–7.18 (m, 6H), 7.18–7.06 (m, 14H), 7.01–6.90 (m, 4H); ^{13}C NMR (75 MHz, CD_2Cl_2): δ 200.91, 155.76, 141.83, 141.73, 140.62, 140.17, 133.96, 133.01, 130.91, 130.60, 130.34, 129.86, 129.61, 129.13, 128.76, 128.39, 128.29, 127.10, 126.99, 125.44; HRMS (MALDI-TOF) *m/z*: $[M]^+$ calcd for $C_{53}H_{36}O$ 688.2766; found 688.2763.

4. ^{1}H NMR and ^{13}C NMR spectra of new compounds

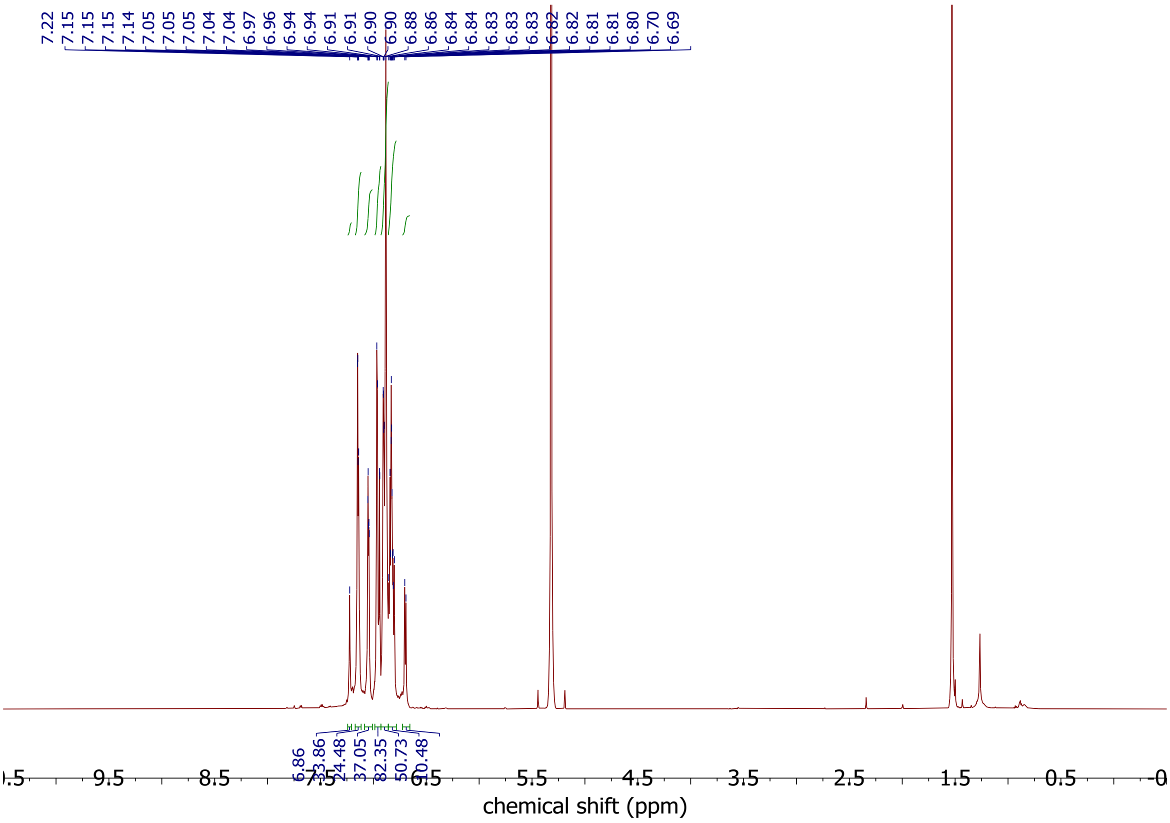

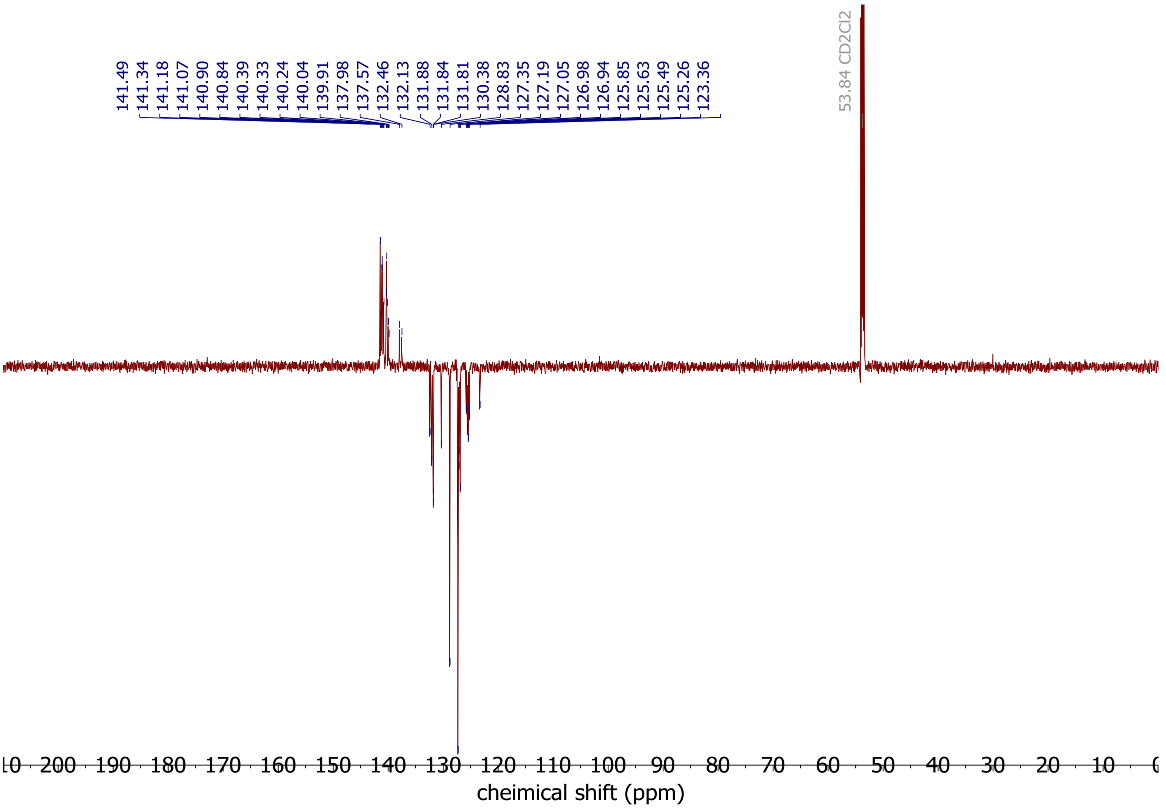

Figure S4. ^{1}H NMR (up, 700 MHz, CD_2Cl_2) and ^{13}C NMR APT spectra (down, 176 MHz, CD_2Cl_2) of **3.**

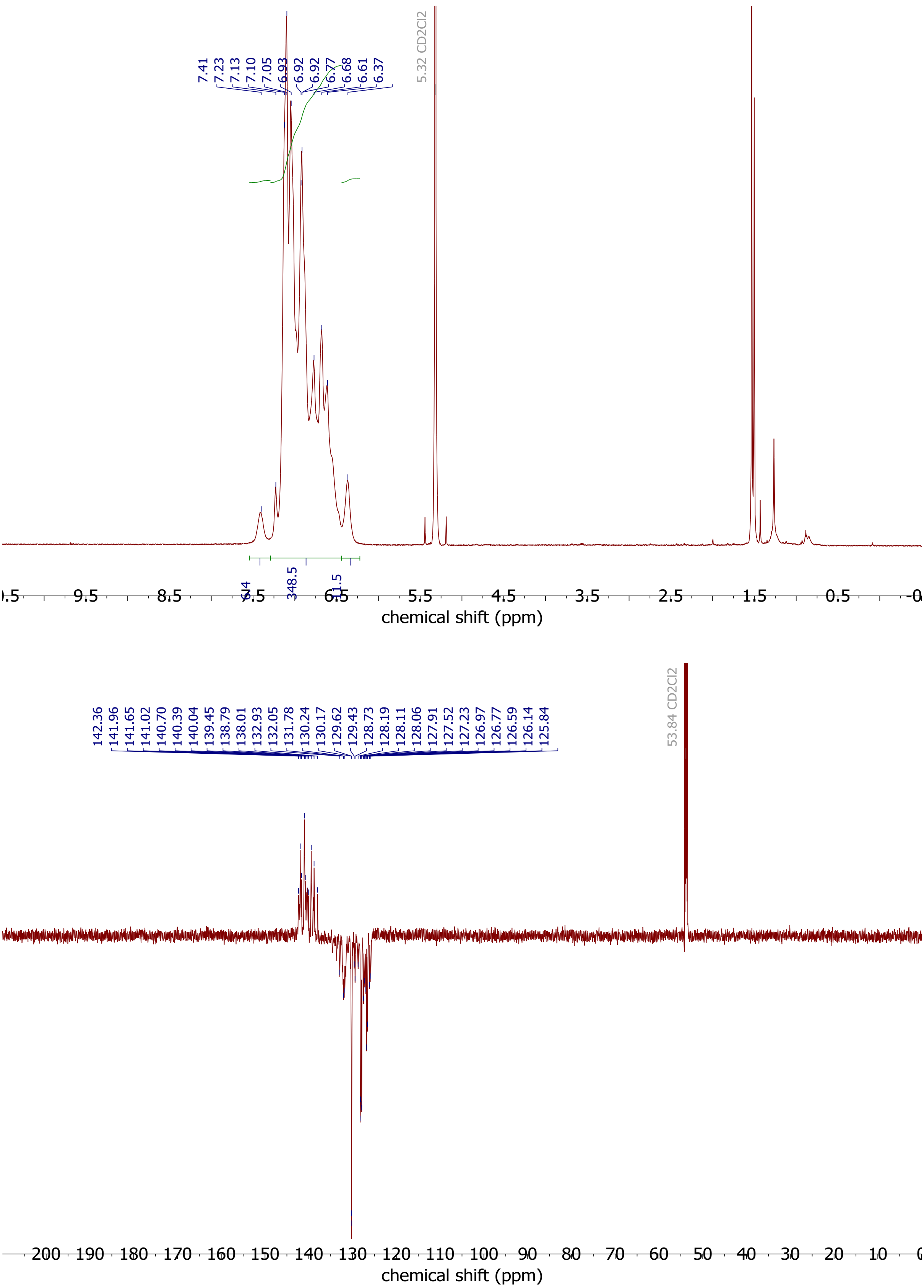

Figure S5. ^{1}H NMR (up, 700 MHz, CD_2Cl_2) and ^{13}C NMR APT spectra (down, 176 MHz, CD_2Cl_2) of **4**.

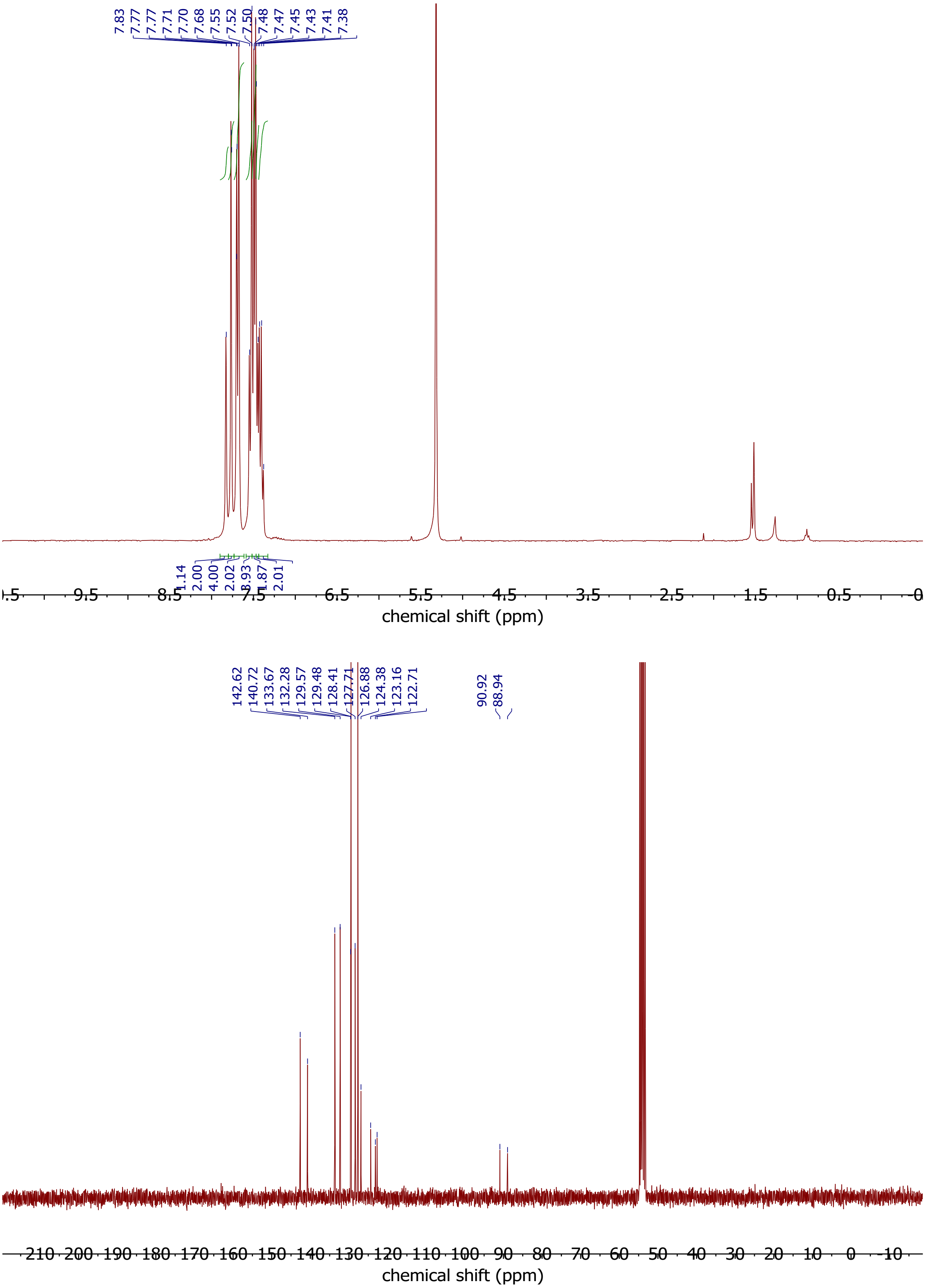

Figure S6. ^{1}H NMR (up, 300 MHz, CD_2Cl_2) and ^{13}C NMR spectra (down, 75 MHz, CD_2Cl_2) of **6**.

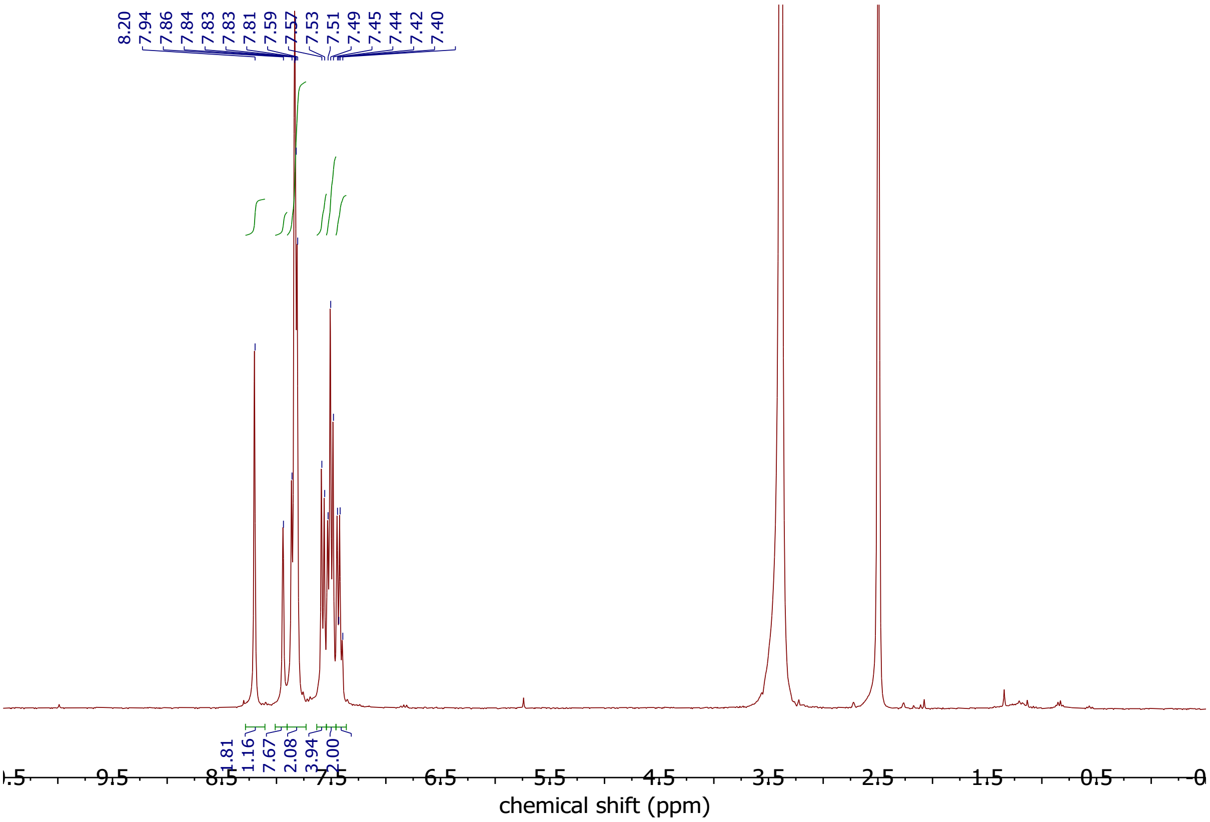

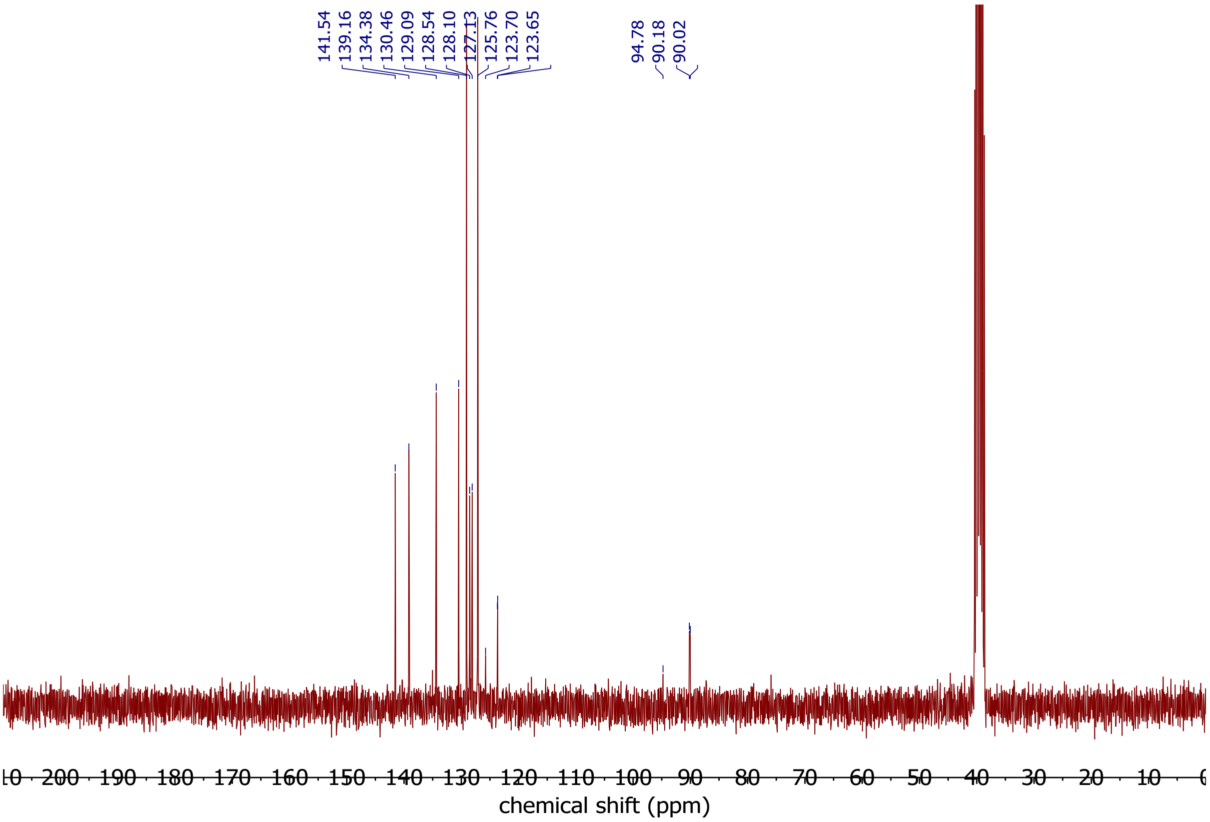

Figure S7. ^{1}H NMR (up, 300 MHz, DMSO-d_6) and ^{13}C NMR spectra (down, 75 MHz, DMSO-d_6) of **7**.

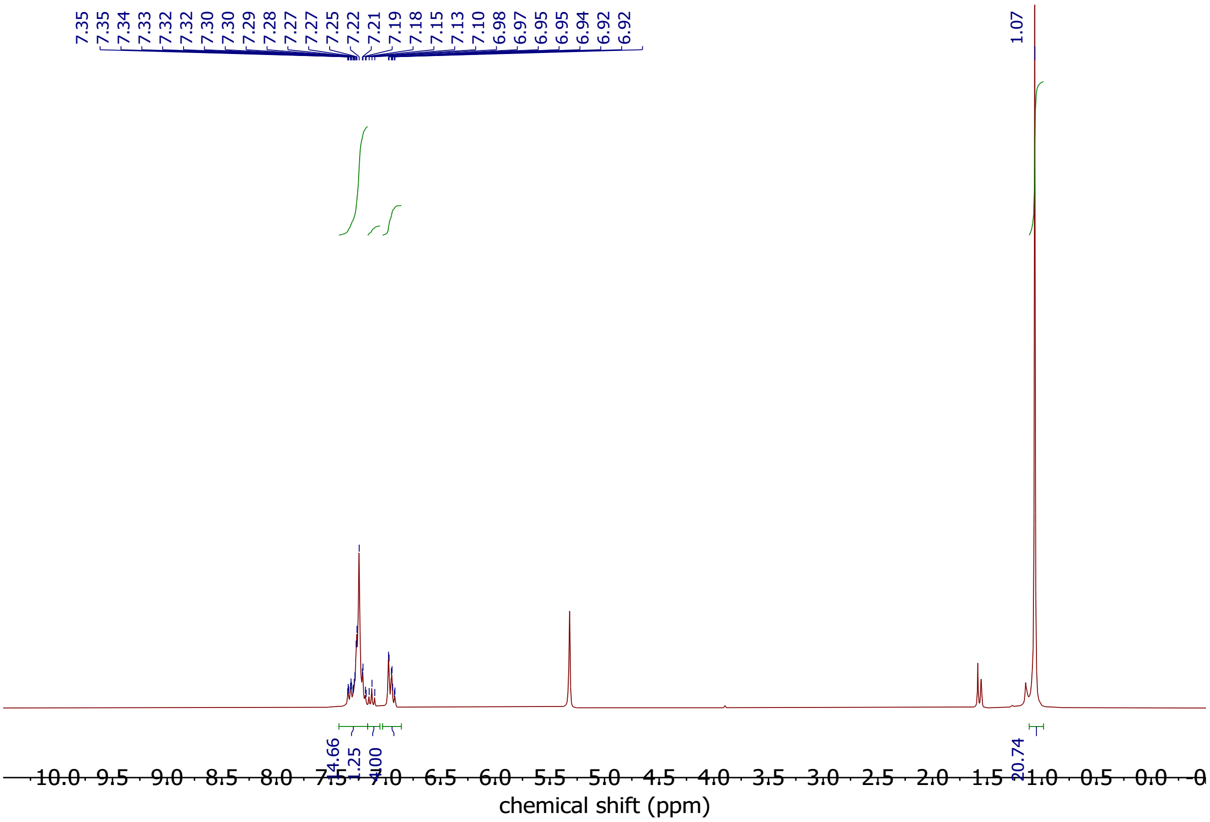

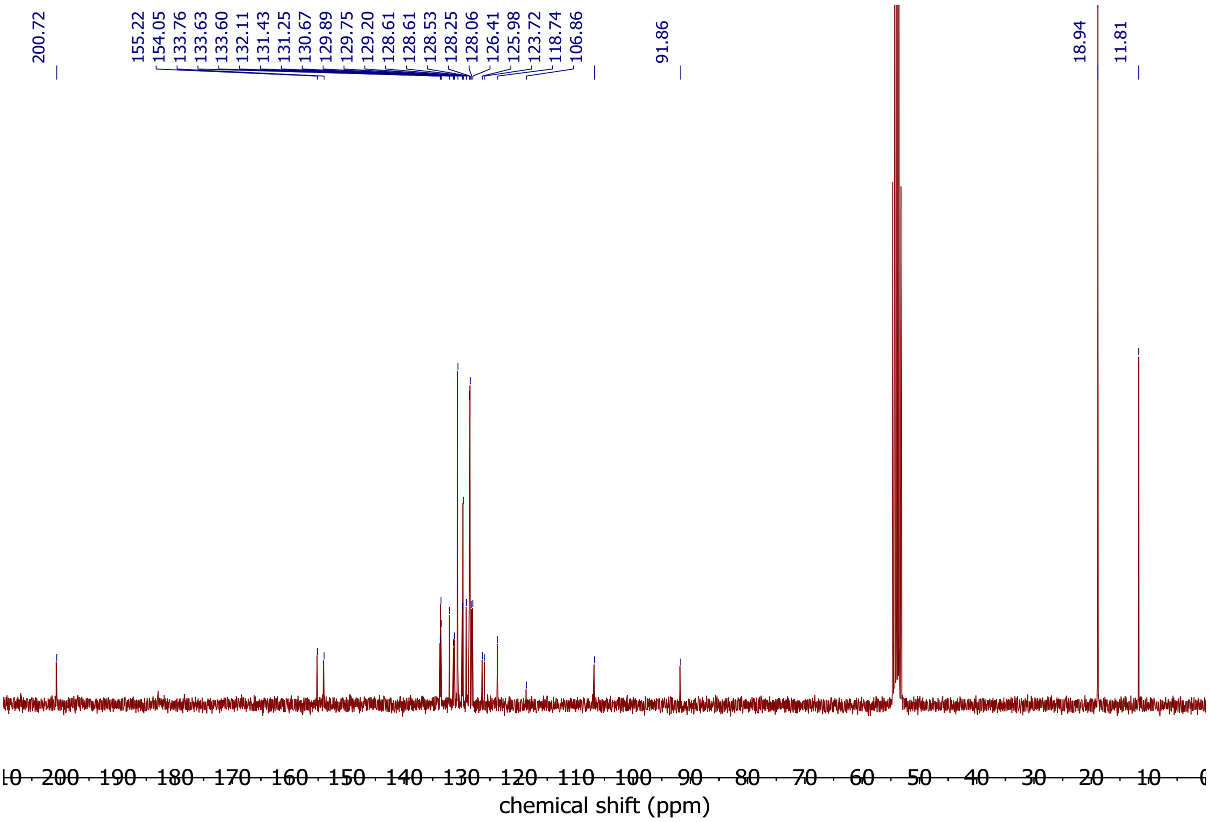

Figure S8. ^{1}H NMR (up, 300 MHz, CD_2Cl_2) and ^{13}C NMR spectra (down, 75 MHz, CD_2Cl_2) of **12**.

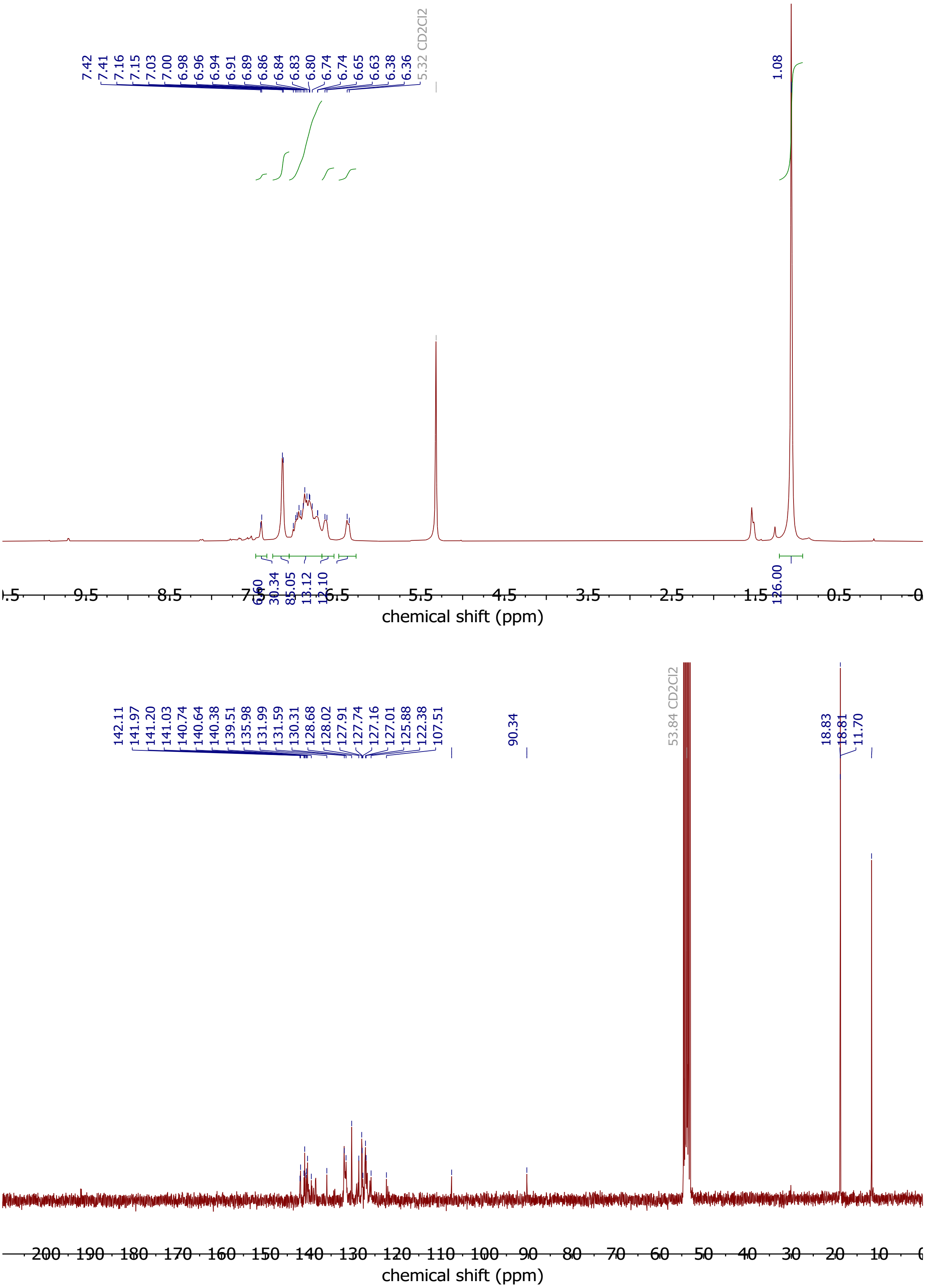

Figure S9. ^{1}H NMR (up, 300 MHz, CD_2Cl_2) and ^{13}C NMR spectra (down, 75 MHz, CD_2Cl_2) of **14**. The relative intensity of the low field impurity signals in the ^{1}H NMR is around 4% to that of aromatic signals of **14**.

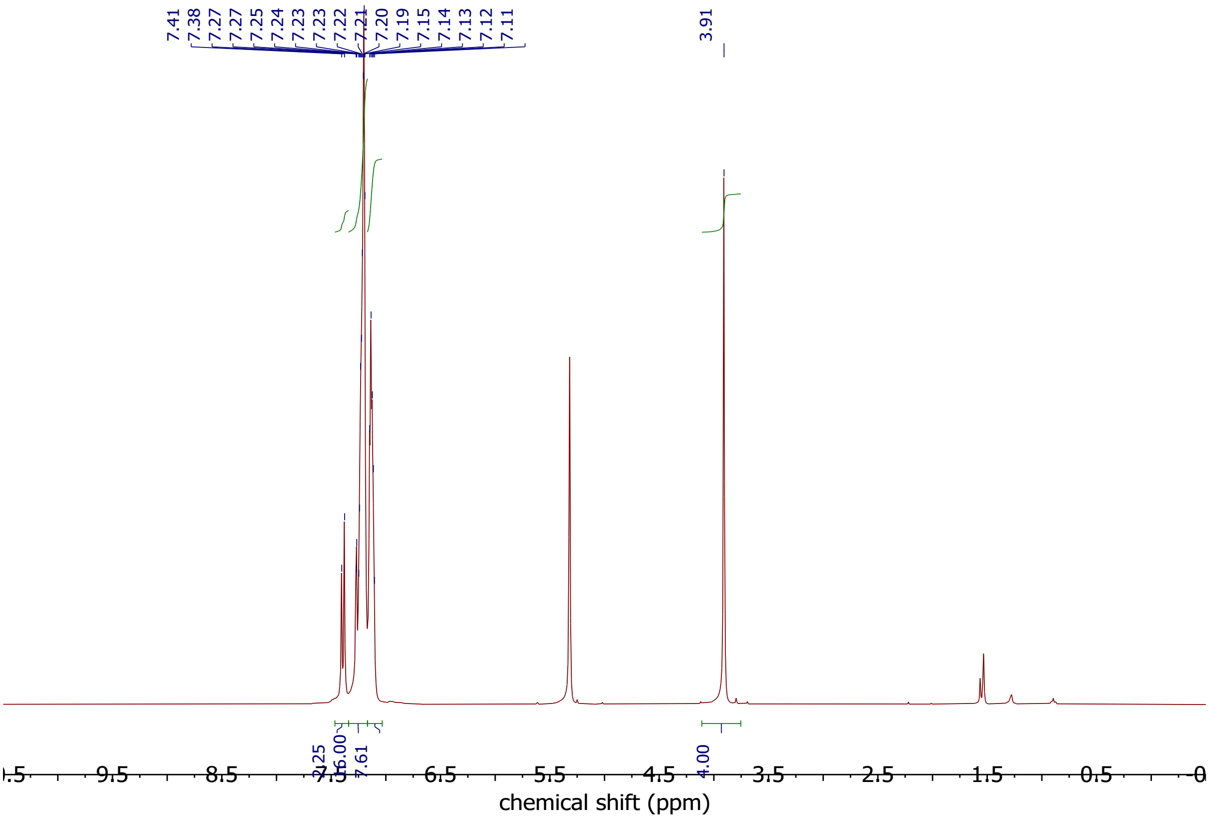

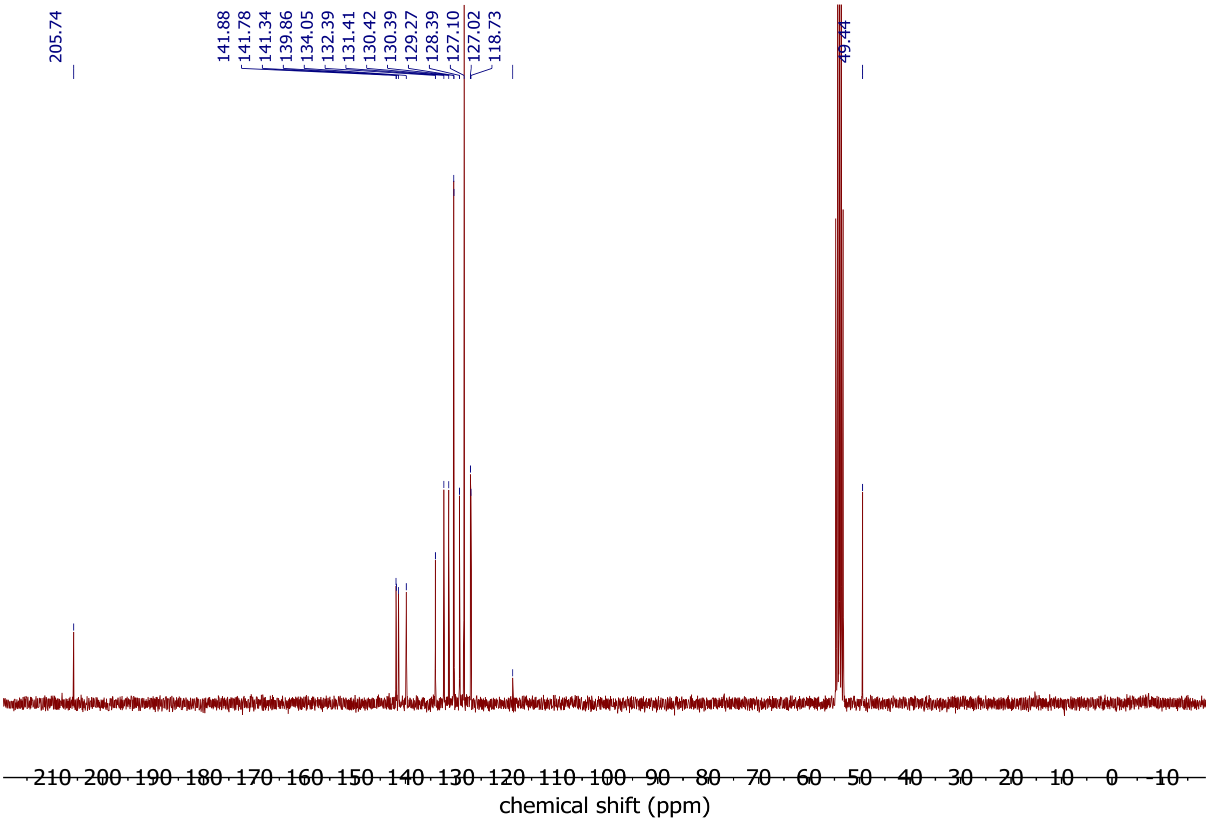

Figure S10. ^{1}H NMR (up, 300 MHz, CD_2Cl_2) and ^{13}C NMR spectra (down, 75 MHz, CD_2Cl_2) of **17**.

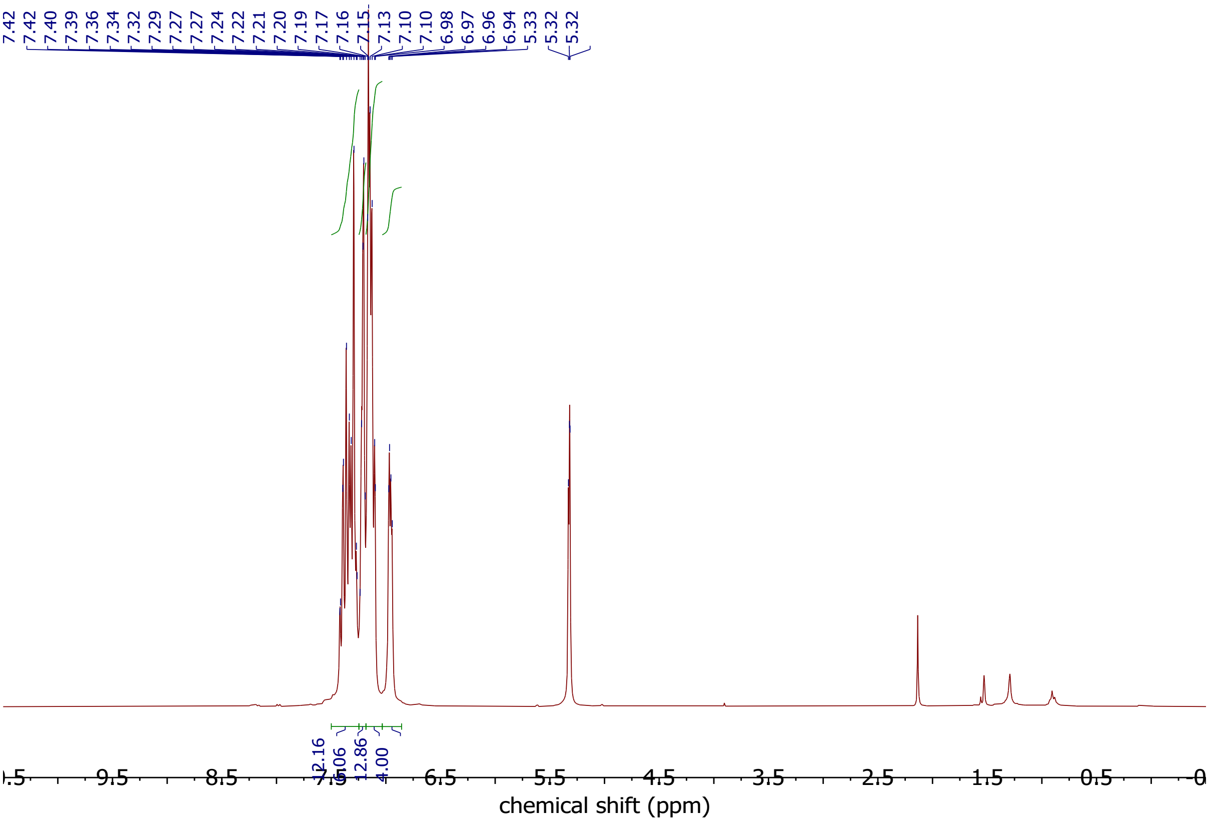

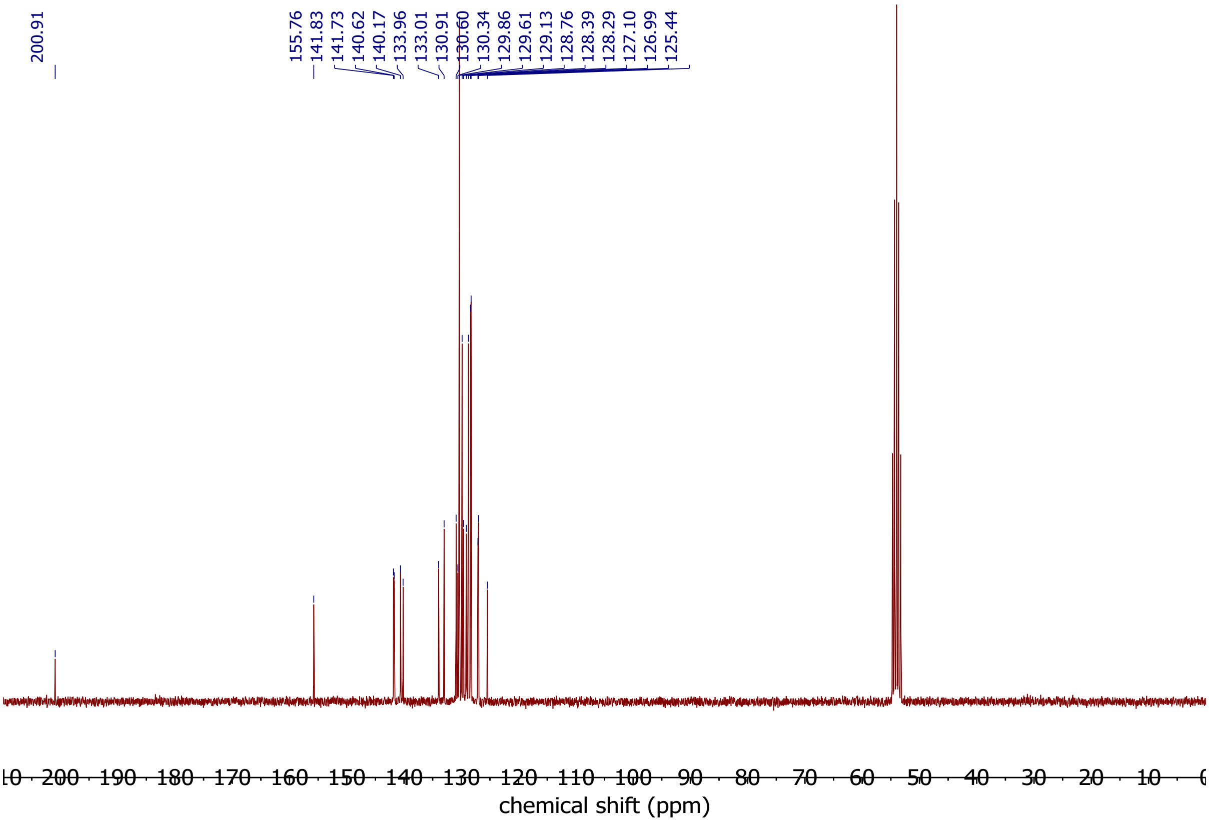

Figure S11. ^{1}H NMR (up, 300 MHz, CD_2Cl_2) and ^{13}C NMR spectra (down, 75 MHz, CD_2Cl_2) of **18**.

5. MALDI-TOF spectra of new compounds

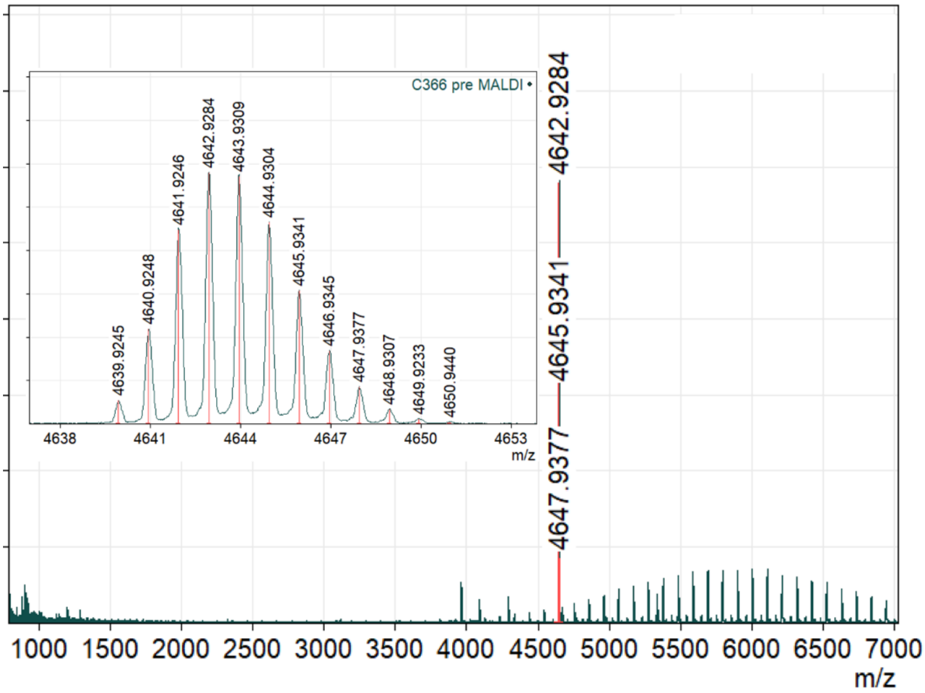

Figure S12. MALDI-TOF spectrum of **3**.

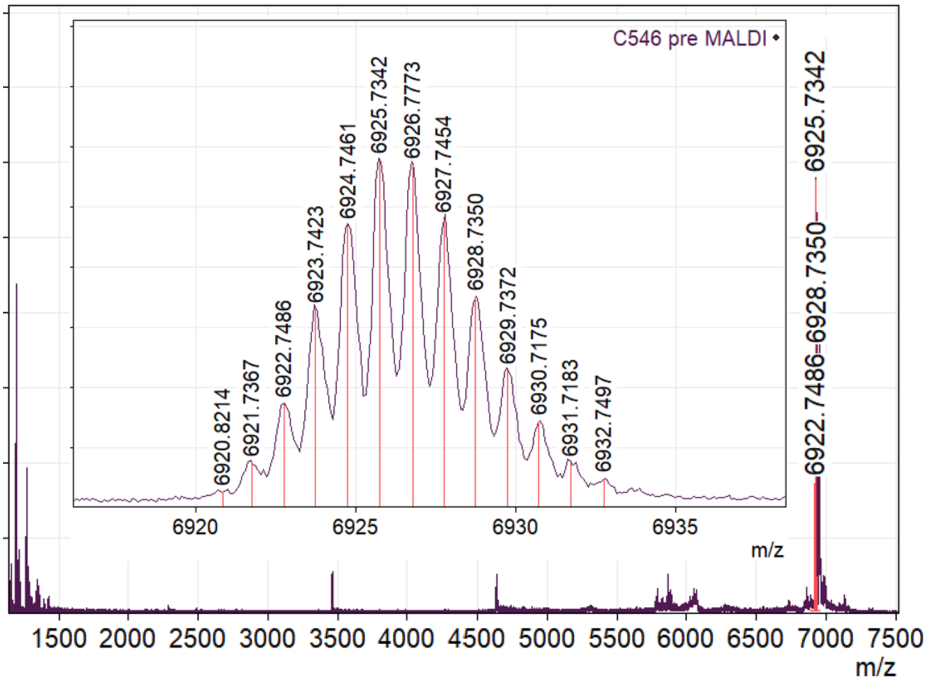

Figure S13. MALDI-TOF spectrum of **4**.

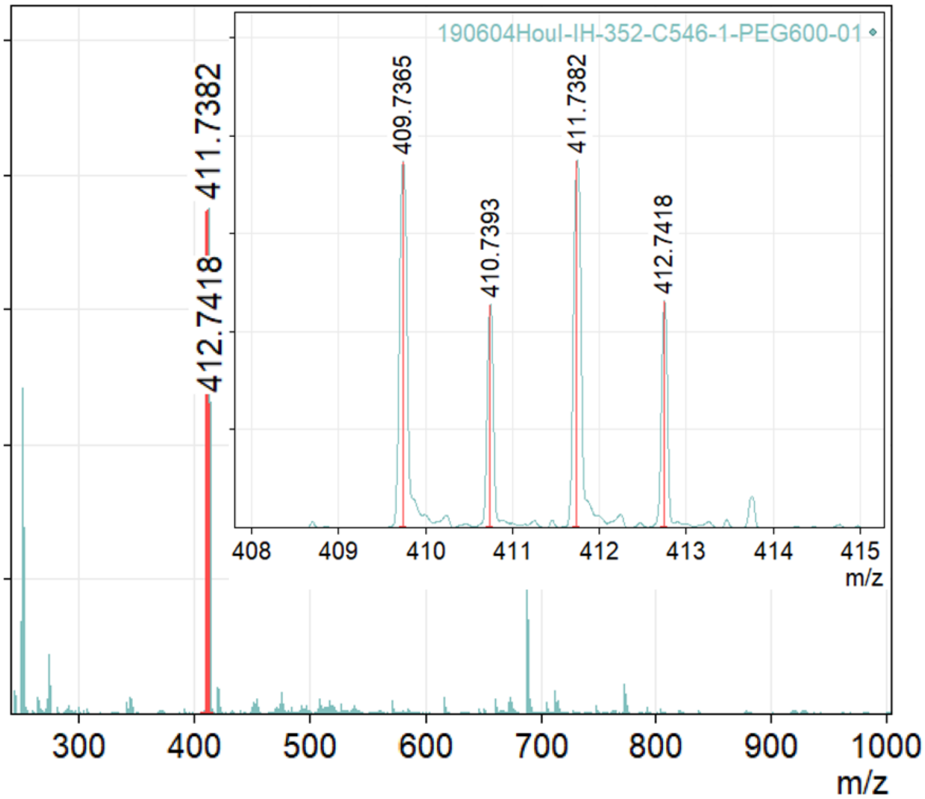

Figure S14. MALDI-TOF spectrum of **6**.

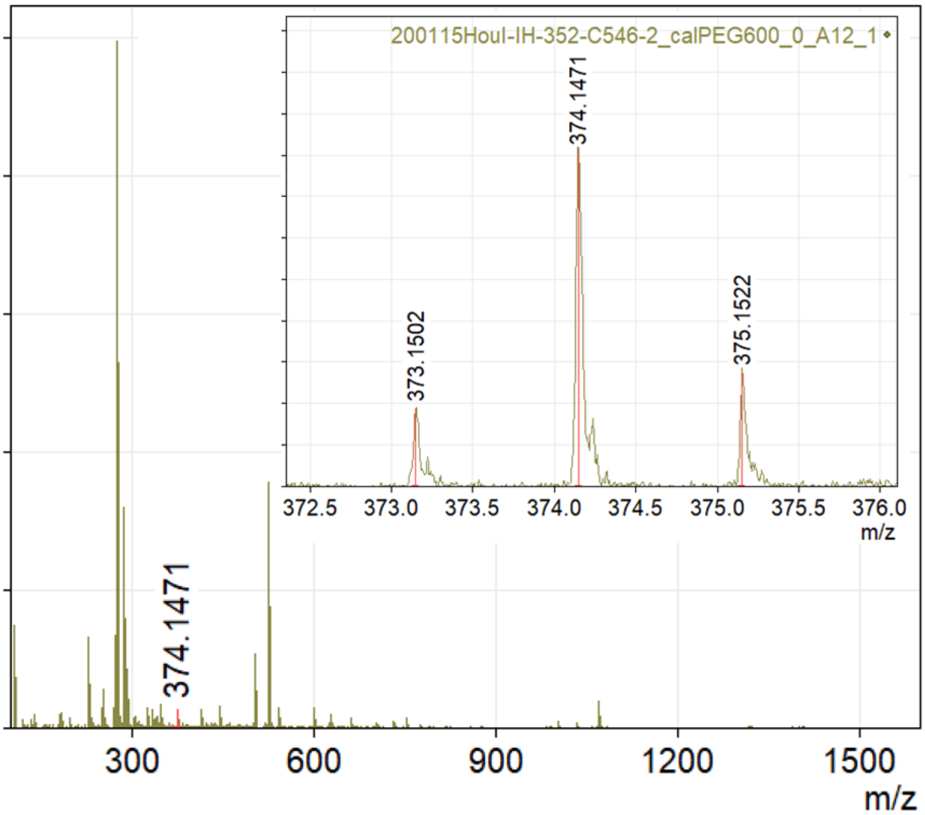

Figure S15. MALDI-TOF spectrum of **7**.

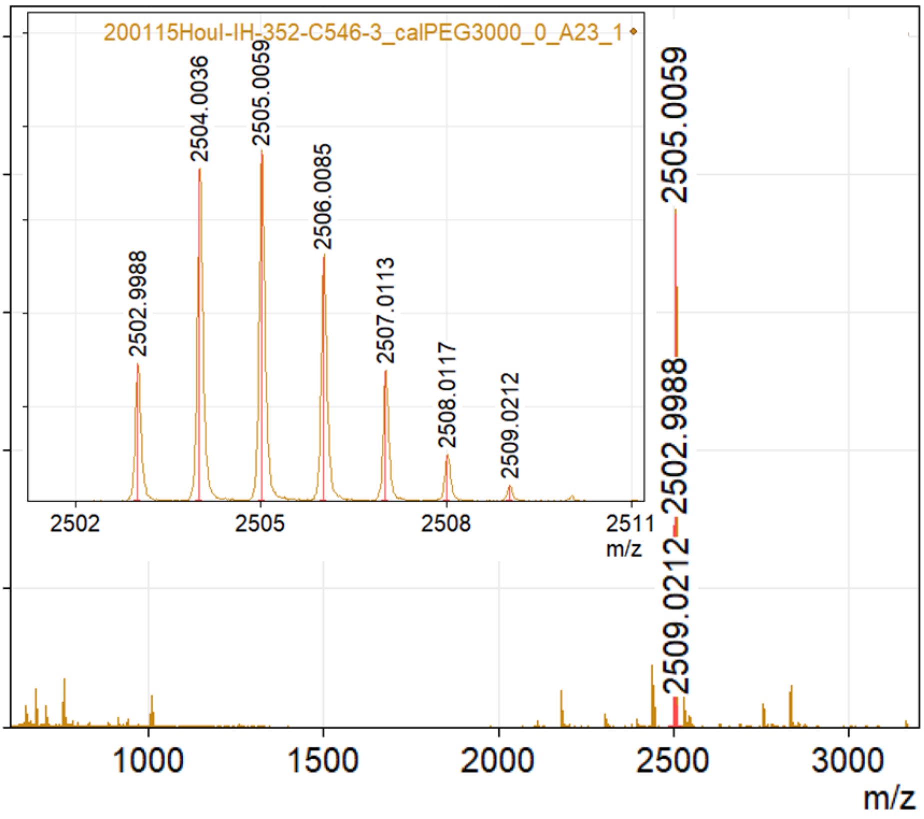

Figure S16. MALDI-TOF spectrum of **9**.

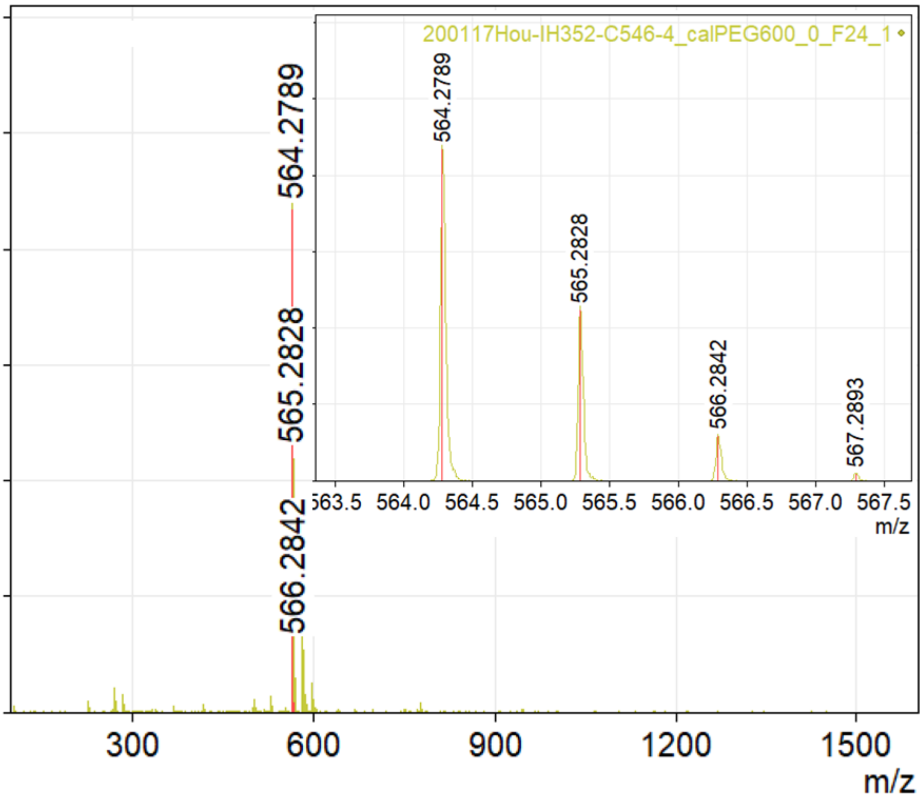

Figure S17. MALDI-TOF spectrum of **12**.

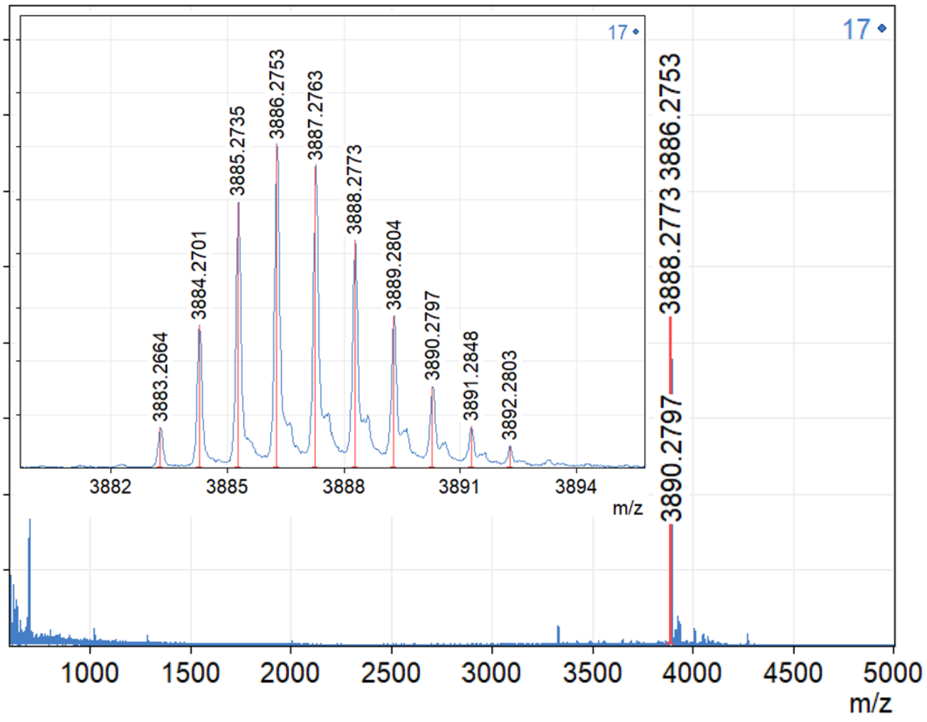

Figure S18. MALDI-TOF spectrum of **14**.

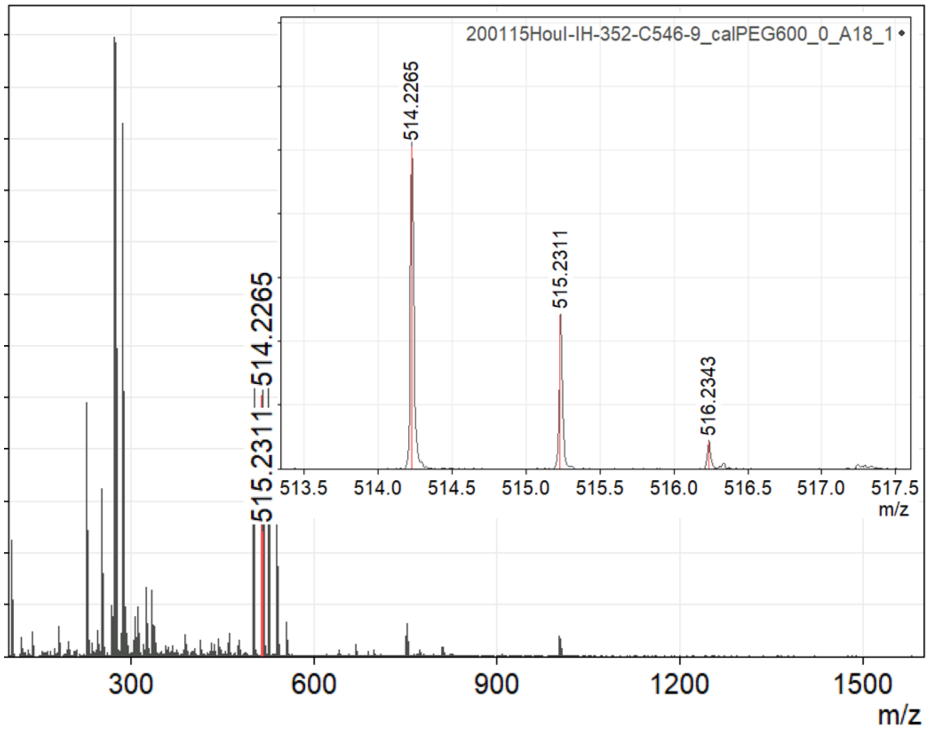

Figure S19. MALDI-TOF spectrum of **17**.

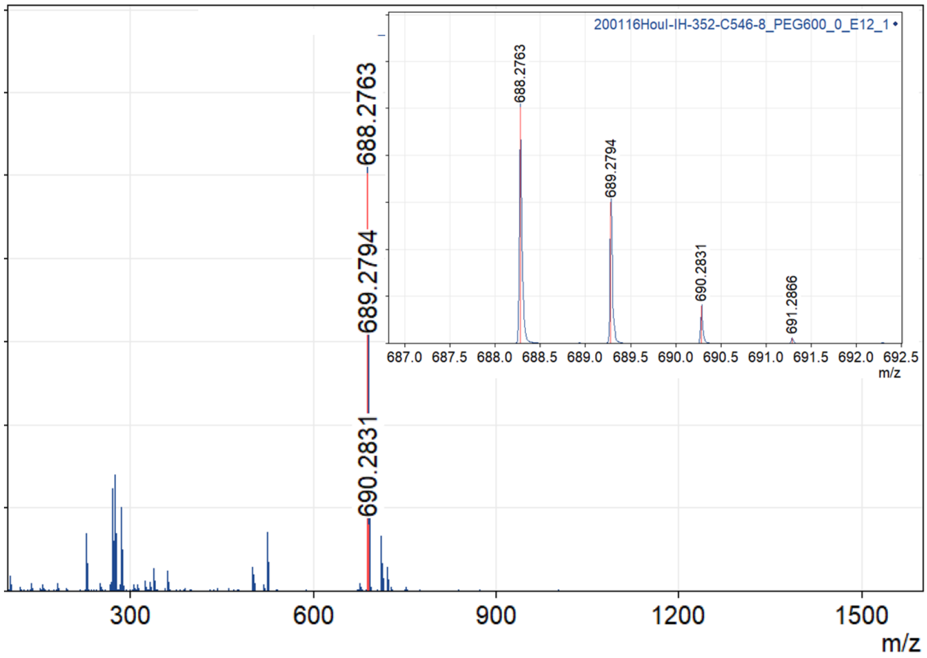

Figure S20. MALDI-TOF spectrum of **18**.

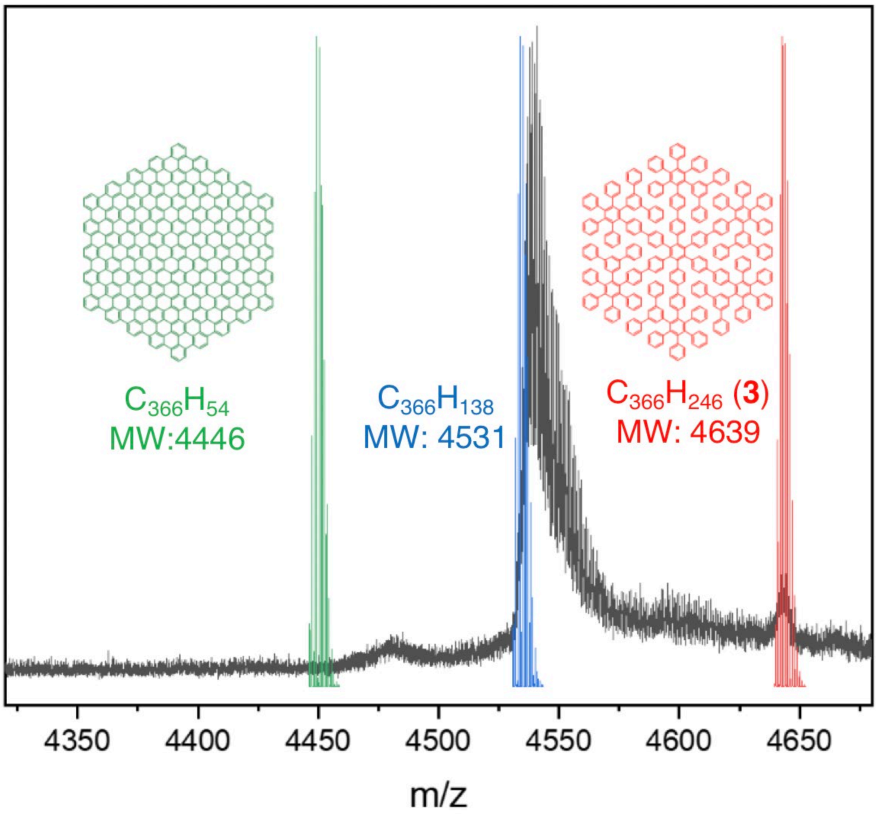

Figure S21. MALDI-TOF spectrum after attempted cyclodehydrogenation of **3** (black line). Condition: 2,3-dichloro-5,6-dicyano-*p*-quinone (DDQ), CF_3SO_3H (TfOH), CH_2Cl_2, room temperature, 12 h. Red, blue, and green lines correspond to theoretical isotope patterns of $C_{366}H_{246}$ (**3**), $C_{366}H_{138}$, and $C_{366}H_{54}$ (NG), respectively. MW: molecular weight.

5. Reference

(1) Zhao, W.; Huang, L.; Guan, Y.; Wulff, W. D. Three-Component Asymmetric Catalytic Ugi Reaction - Concinnity from Diversity by Substrate-Mediated Catalyst Assembly. *Angew. Chemie - Int. Ed.* **2014**, *53* (13), 3436–3441.

(2) Shen, X.; Ho, D. M.; Pascal, R. A. Synthesis of Polyphenylene Dendrimers Related to "Cubic Graphite." *J. Am. Chem. Soc.* **2004**, *126* (18), 5798–5805.

(3) Hu, Y.; Xie, P.; De Corato, M.; Ruini, A.; Zhao, S.; Meggendorfer, F.; Straasø, L. A.; Rondin, L.; Simon, P.; Li, J.; et al. Bandgap Engineering of Graphene Nanoribbons by Control over Structural Distortion. *J. Am. Chem. Soc.* **2018**, *140* (25), 7803–7809.

(4) Narita, A.; Verzhbitskiy, I. A.; Frederickx, W.; Mali, K. S.; Jensen, S. A.; Hansen, M. R.; Bonn, M.; De Feyter, S.; Casiraghi, C.; Feng, X.; et al. Bottom-up Synthesis of Liquid-Phase-Processable Graphene Nanoribbons with near-Infrared Absorption. *ACS Nano* **2014**, *8* (11), 11622–11630.

(5) Narita, A.; Feng, X.; Hernandez, Y.; Jensen, S. A.; Bonn, M.; Yang, H.; Verzhbitskiy, I. A.; Casiraghi, C.; Hansen, M. R.; Koch, A. H. R.; et al. Synthesis of Structurally Well-Defined and Liquid-Phase-Processable Graphene Nanoribbons. *Nat. Chem.* **2014**, *6* (2), 126–132.

(6) Hammer, B. A. G.; Müllen, K. Expanding the Limits of Synthetic Macromolecular Chemistry through Polyphenylene Dendrimers. *J. Nanoparticle Res.* **2018**, *20* (10), 262.

(7) Hinaut, A.; Pawlak, R.; Meyer, E.; Glatzel, T. Electrospray Deposition of Organic Molecules on Bulk Insulator Surfaces. *Beilstein J. Nanotechnol.* **2015**, *6* (1), 1927–1934.

(8) the value is reasonably precise considering the intrinsic difficulty on the acquiring of high resolution MS spectra of larger dendritic PPs. Detailed discussion can be found in (a) C. G. Clark, R. J. Wenzel, E. V. Andreitchenko, W. Steffen, R. Zenobi, K. Müllen, *J. Am. Chem. Soc.* **2007**, *129*, 3292–3301; (b) T.-T.-T. Nguyen, M. Baumgarten, A. Rouhanipour, H. J. Räder, I. Lieberwirth, K. Müllen, *J. Am. Chem. Soc.* **2013**, *135*, 4183–4186; (c) H. J. Räder, T.-T.-T. Nguyen, K. Müllen, *Macromolecules* **2014**, *47*, 1240–1248.
